# Supplementary figures and images for: Altered GC- and AT-biased genotypes of Ophiocordyceps sinensis in the stromal fertile portions and ascospores of natural Cordyceps sinensis
Source: PLoS One. 2023 Jun 8;18(6):e0286865. doi: 10.1371/journal.pone.0286865 (PMC10249794; doi:10.1371/journal.pone.0286865)

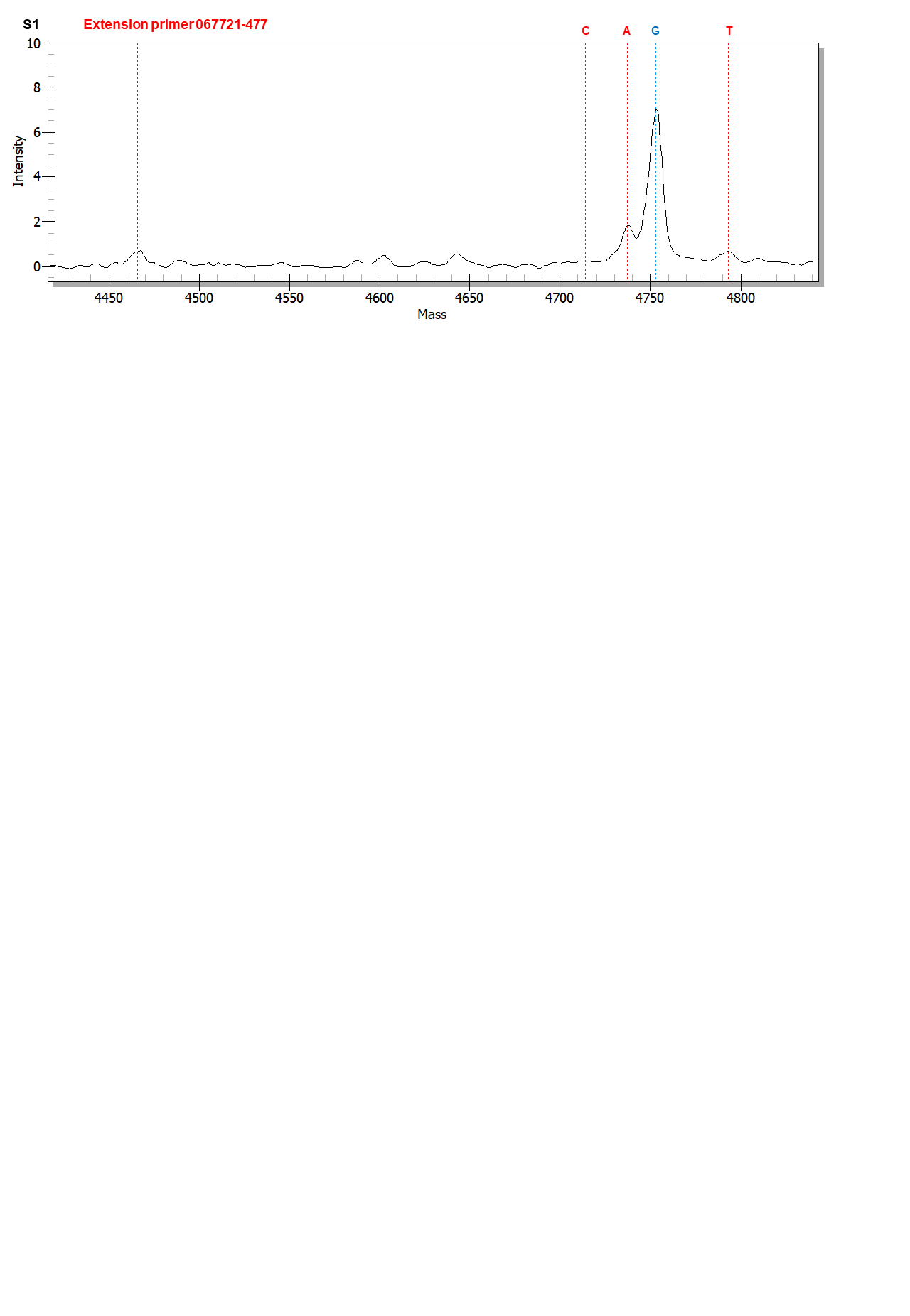

Supplement: S1 Fig — The extension reaction proceeded from extension primer 067721–477 toward the SNP at position 477 in the AB067721 sequence (cf. Fig 1 for the location). In the allelic peaks, “C” denotes extension of the primer with an extended cytosine; “A” indicates an extended adenine, “G” represents an extended guanine, and “T” refers to an extended thymine. (TIF) [file pone.0286865.s001.tif]

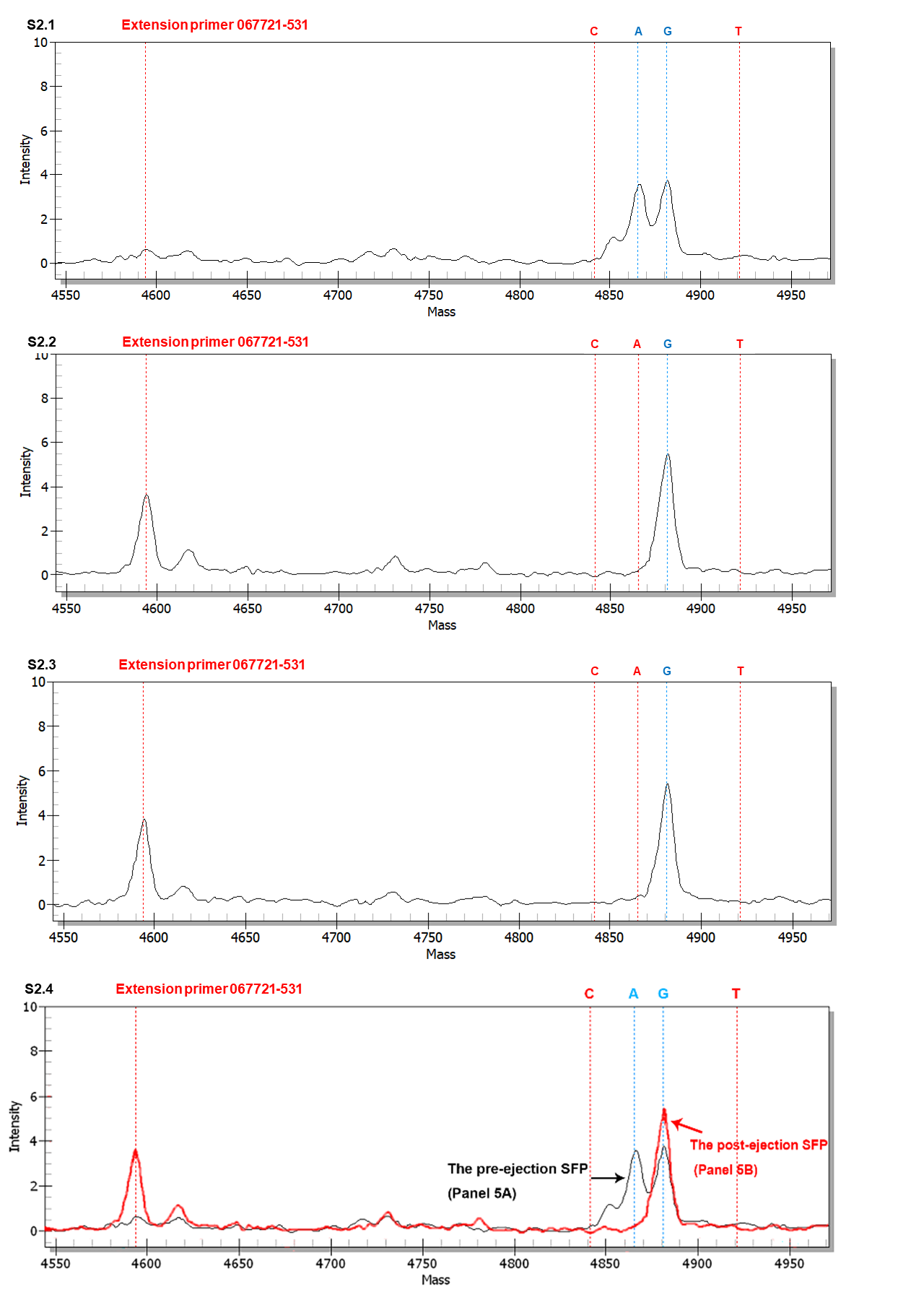

Supplement: S2 Fig — The extension primer 067721–531 was extended to the SNP at position 531 in the AB067721 sequence (cf. Fig 1 for the location). The allele peaks are marked: “C” denotes the primer with an extended cytosine; “A” indicates an extended adenine, “G” represents an extended guanine, and “T” refers to an extended thymine. Panel S2.1 shows the mass spectrum for the SFP before ascospore ejection. Panel S2.2 shows the mass spectrum for the SFP after ascospore ejection. Panel S2.3 shows the mass spectrum for the SFP that failed to develop and eject the ascospore. Panel S2.4 shows the overlapping mass spectra for Panels S2.1 (black tracing) and S2.2 (red tracing) with alignment of the horizontal and vertical axes of the panels. (TIF) [file pone.0286865.s002.tif]

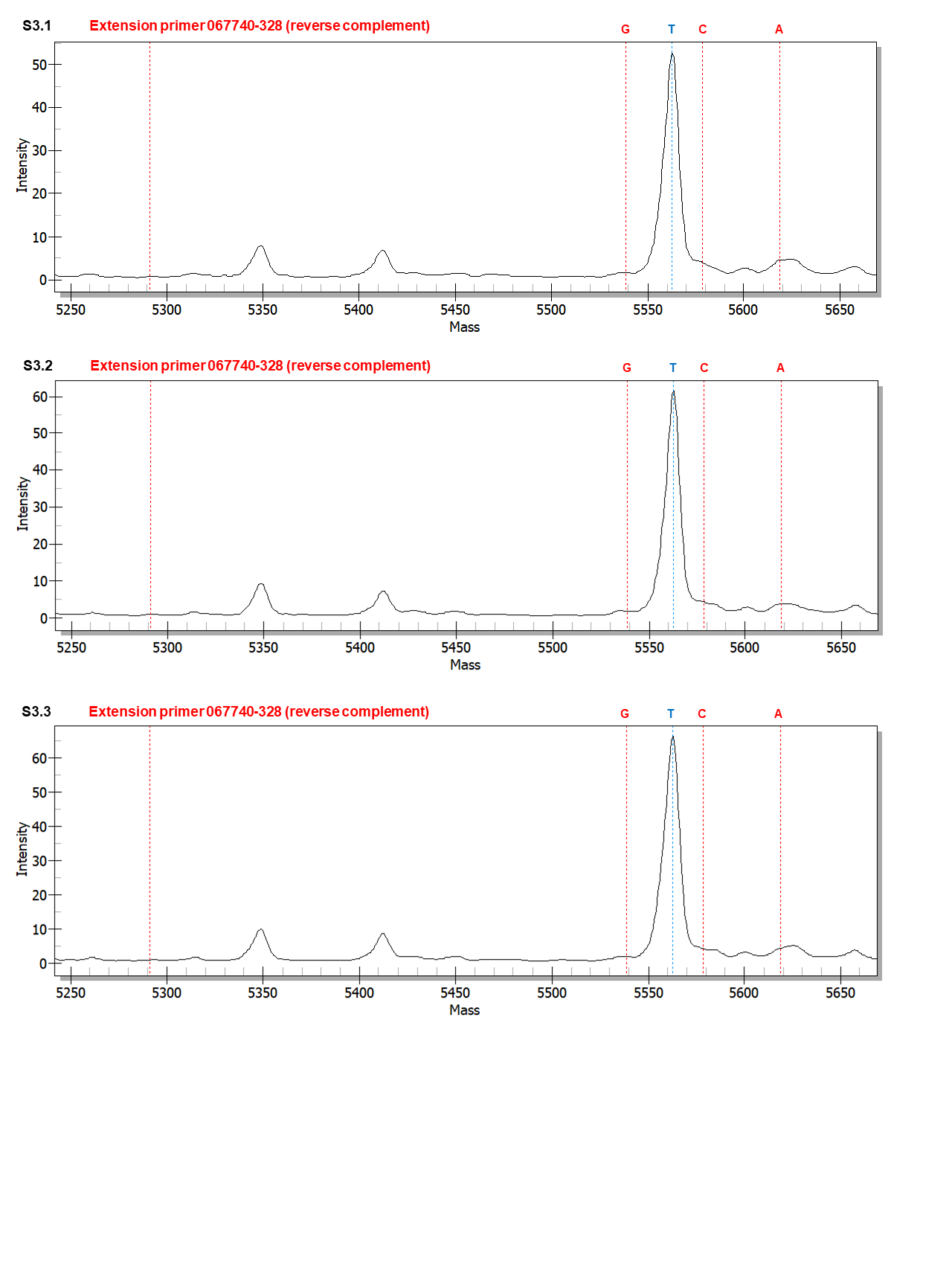

Supplement: S3 Fig — The extension reaction proceeded from extension primer 067740–328 (reverse complement) toward the SNP at position 328 in the AB067740 sequence (cf. Fig 1 for the location). In the allelic peaks, “G” represents the primer with an extended reverse complement cytosine (guanine in the sense chain; cf. Fig 1); “T” represents an extended reverse complement adenine (thymine in the sense chain); “C” denotes an extended reverse complement guanine (cytosine in the sense chain); and “A” indicates an extended reverse complement thymine (adenine in the sense chain). Panel S3.1 shows the mass spectrum of the pre-ejection SFP. Panel S3.2 shows the mass spectrum of the post-ejection SFP. Panel S3.3 shows the mass spectrum of the SFP of developmental failure. (TIF) [file pone.0286865.s003.tif]

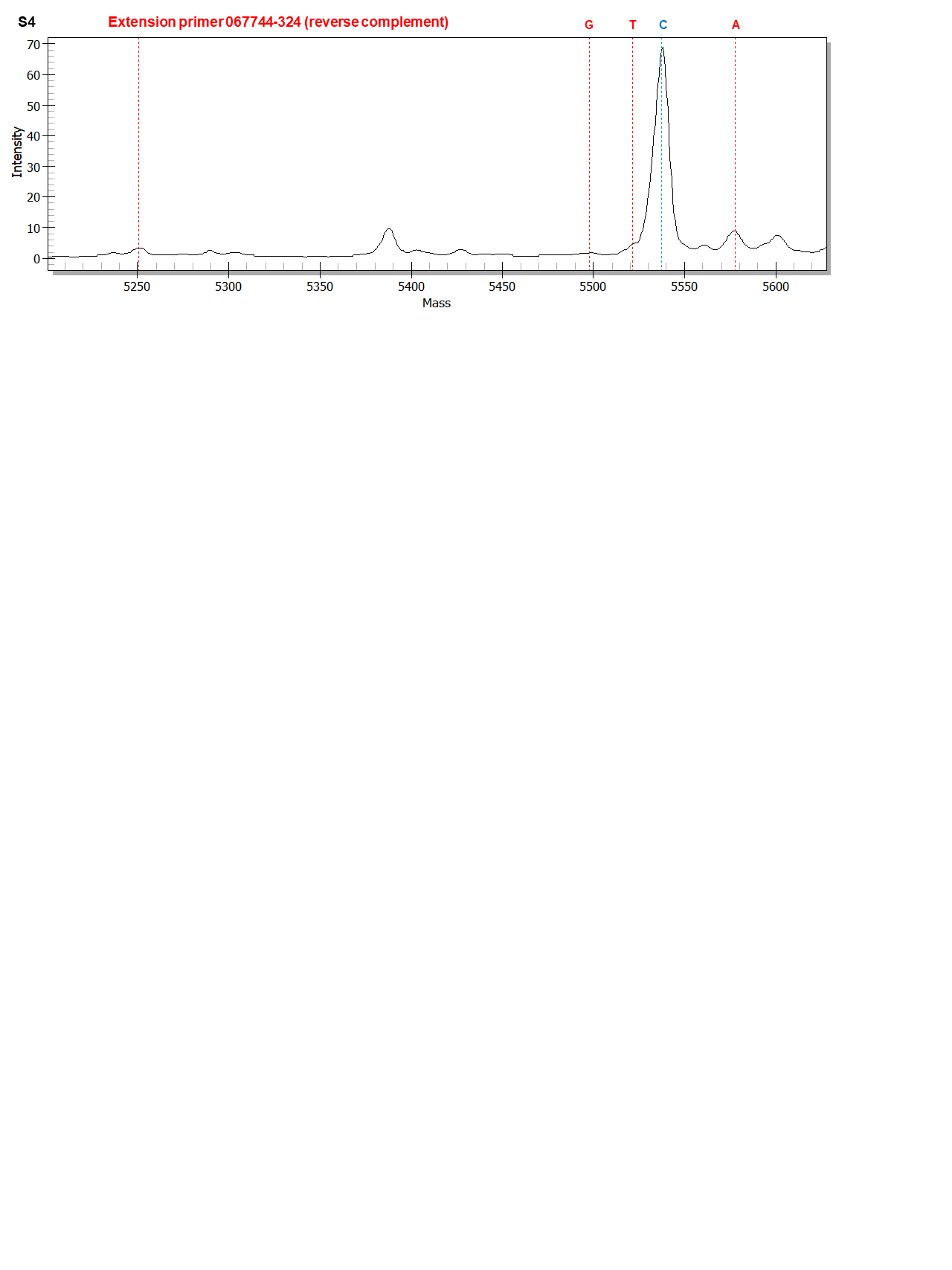

Supplement: S4 Fig — The extension reaction proceeded from extension primer 067744–324 (reverse complement) toward the SNP at position 324 in the AB067740 sequence (cf. Fig 1 for the location). In the allelic peaks, “G” represents the primer with an extended reverse complement cytosine (guanine in the sense chain; cf. Fig 1); “T” represents an extended reverse complement adenine (thymine in the sense chain); “C” denotes an extended reverse complement guanine (cytosine in sense chain); and “A” indicates an extended reverse complement thymine (adenine in the sense chain). This shows the mass spectrum of the post-ejection SFP. (TIF) [file pone.0286865.s004.tif]

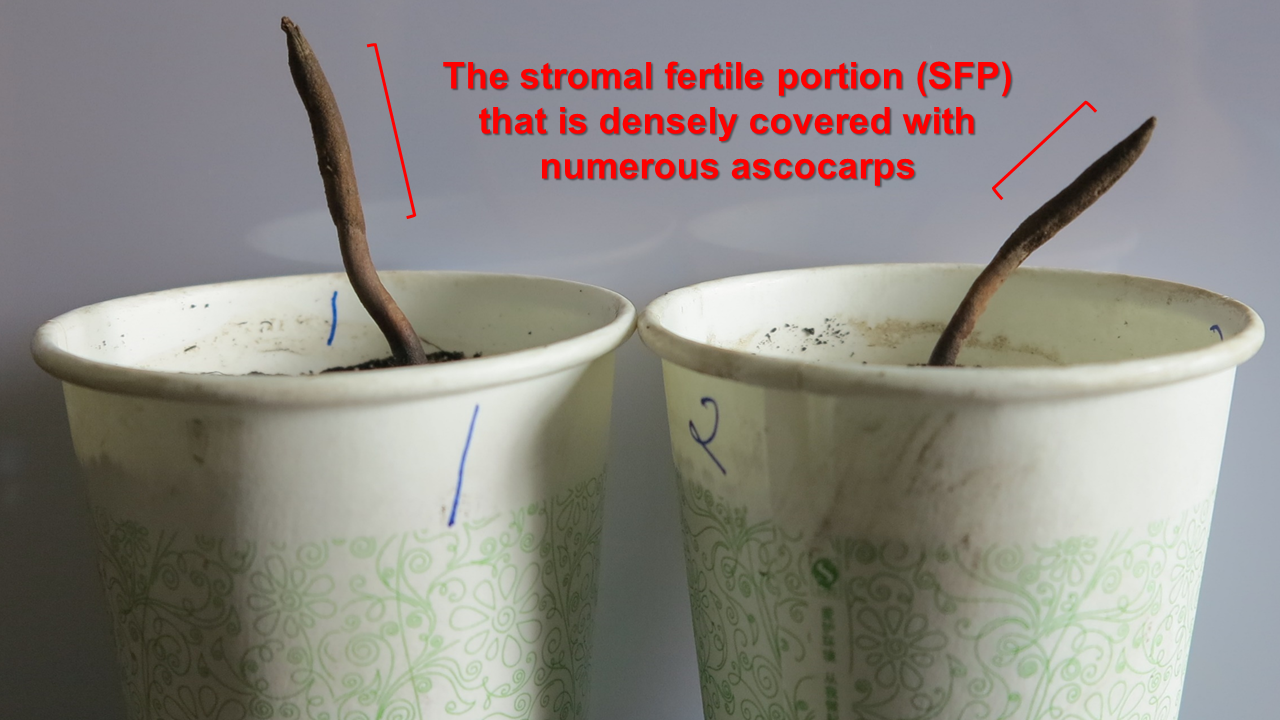

Supplement: S5 Fig — Mature Cordyceps sinensis specimens were cultivated in our Xining laboratory (altitude of 2,254 m) (SR1(A) Fig). The fully ejected ascospores were collected using double layers of autoclaved weighing papers (SR1(B) Fig). Numerous semi-ejected ascospores adhering to the outer surface of an ascus (SR1(C) Fig) during the massive ejection of ascospores. The stromal fertile portion (SFP) densely covered with numerous ascocarps is labeled with “]”. (ZIP) [file pone.0286865.s011.Zip › SR1(A)_Fig.tif]

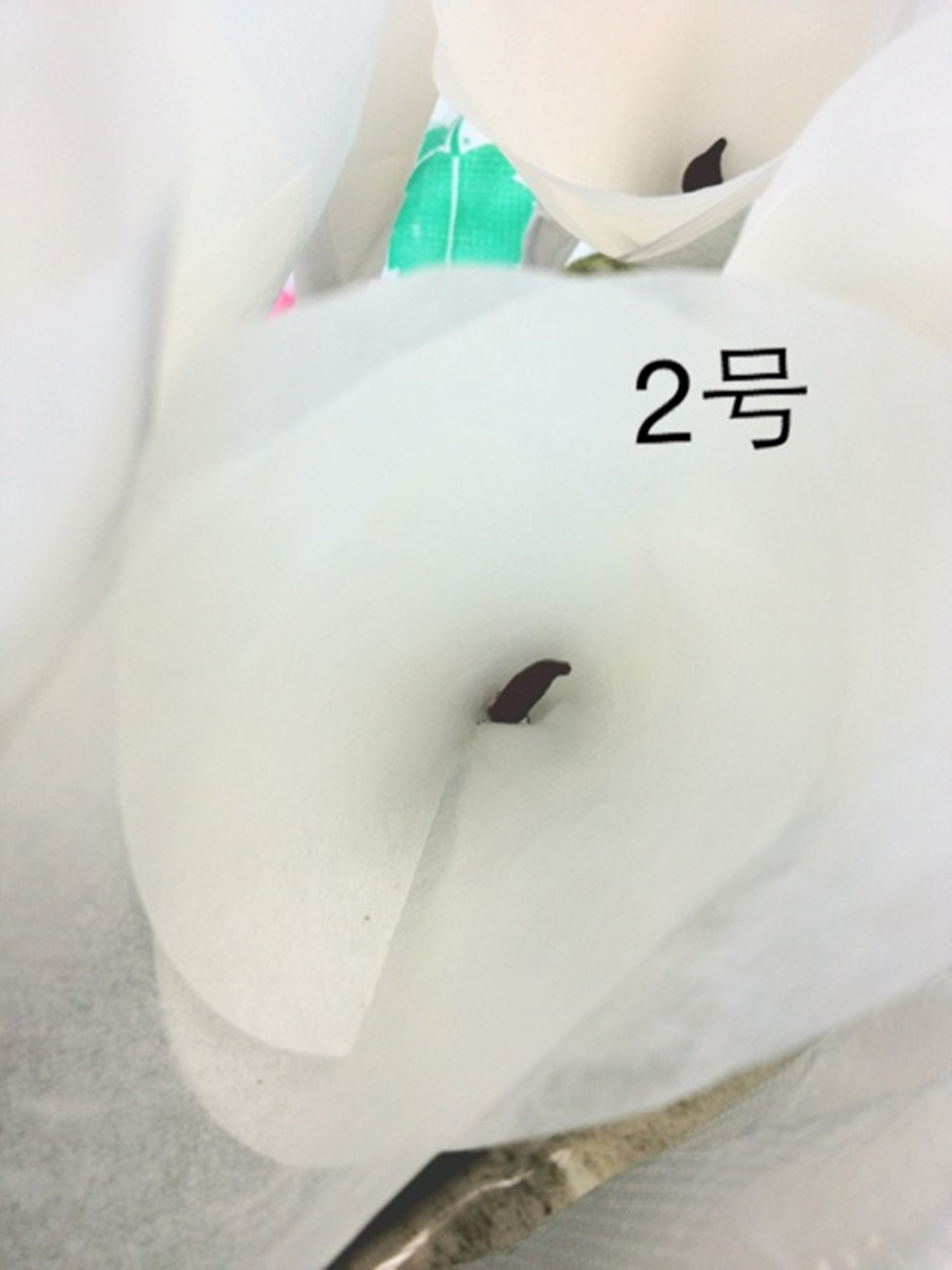

Supplement: S5 Fig — Mature Cordyceps sinensis specimens were cultivated in our Xining laboratory (altitude of 2,254 m) (SR1(A) Fig). The fully ejected ascospores were collected using double layers of autoclaved weighing papers (SR1(B) Fig). Numerous semi-ejected ascospores adhering to the outer surface of an ascus (SR1(C) Fig) during the massive ejection of ascospores. The stromal fertile portion (SFP) densely covered with numerous ascocarps is labeled with “]”. (ZIP) [file pone.0286865.s011.Zip › SR1(B)_Fig.tif]

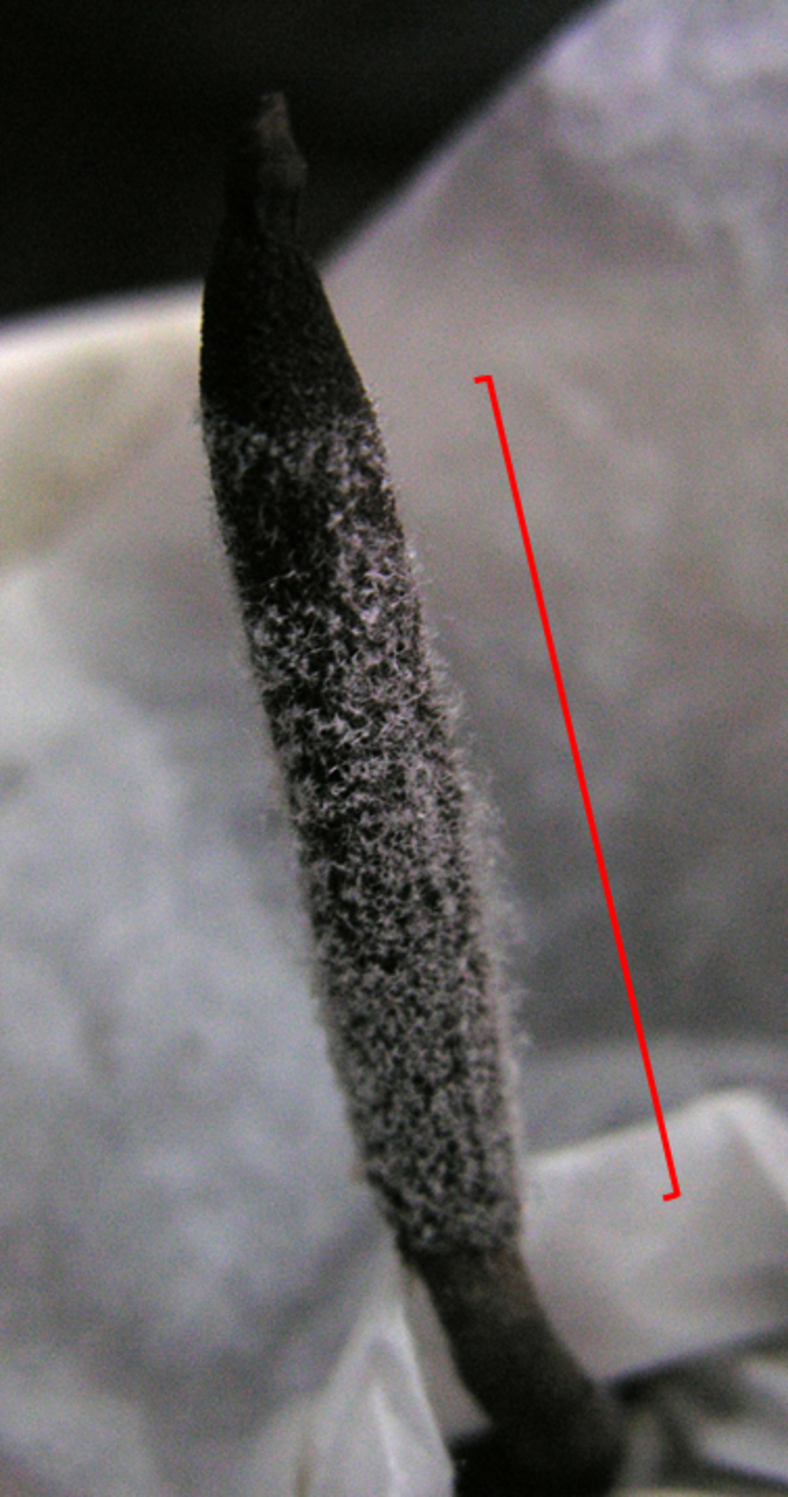

Supplement: S5 Fig — Mature Cordyceps sinensis specimens were cultivated in our Xining laboratory (altitude of 2,254 m) (SR1(A) Fig). The fully ejected ascospores were collected using double layers of autoclaved weighing papers (SR1(B) Fig). Numerous semi-ejected ascospores adhering to the outer surface of an ascus (SR1(C) Fig) during the massive ejection of ascospores. The stromal fertile portion (SFP) densely covered with numerous ascocarps is labeled with “]”. (ZIP) [file pone.0286865.s011.Zip › SR1(C)_Fig .tif]
