# Supplementary material for: Altered GC- and AT-biased genotypes of Ophiocordyceps sinensis in the stromal fertile portions and ascospores of natural Cordyceps sinensis
Source: PLoS One. 2023 Jun 8;18(6):e0286865. doi: 10.1371/journal.pone.0286865 (PMC10249794; doi:10.1371/journal.pone.0286865)
Supplement: S1 Table — (DOCX) [file pone.0286865.s005.docx]

**S1 Table.** **Percent contents of GC and AT bases in the ITS1-5.8S-ITS2 sequences of multiple genotypes of *O. sinensis*.**

|  | Genotype | Representative sequence | % GC content | % AT content |
| --- | --- | --- | --- | --- |
| GC-biased | #1 | AB067721 | 63.3% | 36.7% |
|  | #2 | MG770309 | 58.1% | 41.9% |
|  | #3 | HM595984 | 63.3% | 36.7% |
|  | #7 | AJ488254 | 65.3% | 34.7% |
|  | #8 | GU246286 | 59.5% | 40.5% |
|  | #9 | GU246288 | 64.4% | 35.6% |
|  | #10 | GU246287 | 59.8% | 40.2% |
|  | #11 | JQ695935 | 56.3% | 43.7% |
|  | #12 | GU246296 | 63.7% | 36.3% |
|  | #13 | KT339190 | 58.5% | 41.5% |
|  | #14 | KT339178 | 58.1% | 41.9% |
| AT-biased | #4 | AB067744 | 52.2% | 47.8% |
|  | #5 | AB067740 | 48.1% | 51.9% |
|  | #6 | EU555436 | 51.3% | 48.7% |
|  | #15 | KT232017 | 53.1% | 46.9% |
|  | #16 | KT232019 | 50.9% | 49.1% |
|  | #17 | KT232010 | 51.2% | 48.8% |
