# Supplementary material for: Altered GC- and AT-biased genotypes of Ophiocordyceps sinensis in the stromal fertile portions and ascospores of natural Cordyceps sinensis
Source: PLoS One. 2023 Jun 8;18(6):e0286865. doi: 10.1371/journal.pone.0286865 (PMC10249794; doi:10.1371/journal.pone.0286865)
Supplement: S2 Table — (DOCX) [file pone.0286865.s006.docx]

**S2 Table.** **GenBank accession numbers that are hyperlinked to the GenBank database****, *H. sinensis* strain, *O. sinensis* isolate, amplicon clone information, and sequence ranges for the 52 ITS sequences analyzed phylogenetically in Fig 2.**

| GenBank Accession# | *H. sinensis* strain | *O. sinensis* isolate or amplicon clone | Sequence range |
| --- | --- | --- | --- |
| [LWBQ01000008](https://www.ncbi.nlm.nih.gov/nuccore/LWBQ01000008) | ZJB12195 |  | 991797→992287 |
| [NGJJ01000753](https://www.ncbi.nlm.nih.gov/nuccore/NGJJ01000753) | CC1406-203 |  | 14824→14113 |
| [NGJJ01000852](https://www.ncbi.nlm.nih.gov/nuccore/NGJJ01000852) | CC1406-203 |  | 10703→11192 |
| [ANOV01021709](https://www.ncbi.nlm.nih.gov/nuccore/ANOV01021709) | Co18 |  | 896→1386 |
| [JAAVMX010000002](https://www.ncbi.nlm.nih.gov/nuccore/JAAVMX010000002) | IOZ07 |  | 18688917→18689407 |
| [JAAVMX010000008](https://www.ncbi.nlm.nih.gov/nuccore/JAAVMX010000008) | IOZ07 |  | 1199→1687 |
| [JAAVMX010000008](https://www.ncbi.nlm.nih.gov/nuccore/JAAVMX010000008) | IOZ07 |  | 13823→14313 |
| [JAAVMX010000017](https://www.ncbi.nlm.nih.gov/nuccore/JAAVMX010000017) | IOZ07 |  | 47097→47569 |
| [JAAVMX010000018](https://www.ncbi.nlm.nih.gov/nuccore/JAAVMX010000018) | IOZ07 |  | 700→1186 |
| [JAAVMX010000017](https://www.ncbi.nlm.nih.gov/nuccore/JAAVMX010000017) | IOZ07 |  | 9147→9637 |
| [JAAVMX010000017](https://www.ncbi.nlm.nih.gov/nuccore/JAAVMX010000017) | IOZ07 |  | 21791→22281 |
| [JAAVMX010000018](https://www.ncbi.nlm.nih.gov/nuccore/JAAVMX010000018) | IOZ07 |  | 51467→51958 |
| [JAAVMX010000018](https://www.ncbi.nlm.nih.gov/nuccore/JAAVMX010000018) | IOZ07 |  | 38771→39261 |
| [JAAVMX010000018](https://www.ncbi.nlm.nih.gov/nuccore/JAAVMX010000018) | IOZ07 |  | 26076→26566 |
| [JAAVMX010000018](https://www.ncbi.nlm.nih.gov/nuccore/JAAVMX010000018) | IOZ07 |  | 13381→13871 |
| [JAAVMX010000019](https://www.ncbi.nlm.nih.gov/nuccore/JAAVMX010000019) | IOZ07 |  | 44729→45251 |
| [JAAVMX010000019](https://www.ncbi.nlm.nih.gov/nuccore/JAAVMX010000019) | IOZ07 |  | 332048→32538 |
| [JAAVMX010000019](https://www.ncbi.nlm.nih.gov/nuccore/JAAVMX010000019) | IOZ07 |  | 19404→19894 |
| [LKHE01000582](https://www.ncbi.nlm.nih.gov/nuccore/LKHE01000582) | 1229 |  | 2132→2622 |
| [NGJJ01000796](https://www.ncbi.nlm.nih.gov/nuccore/NGJJ01000796) | CC1406-203 |  | 1514→2004 |
| [NGJJ01000799](https://www.ncbi.nlm.nih.gov/nuccore/NGJJ01000799) | CC1406-203 |  | 1666→17156 |
| [GU246296](https://www.ncbi.nlm.nih.gov/nuccore/GU246296) |  | Isolate ISO-29 | 6→509 |
| [JAAVMX010000017](https://www.ncbi.nlm.nih.gov/nuccore/JAAVMX010000017) | IOZ07 |  | 34435→34925 |
| [KJ175199](https://www.ncbi.nlm.nih.gov/nuccore/KJ175199) | Haplotype 7 |  | 5→492 |
| [HM595984](https://www.ncbi.nlm.nih.gov/nuccore/HM595984) | CUHK:CDC2-2 |  | 33→519 |
| [AB067721](https://www.ncbi.nlm.nih.gov/nuccore/AB067721) |  | Isolate GYOKUJU | 59→549 |
| [AB067715](https://www.ncbi.nlm.nih.gov/nuccore/AB067715) |  | Isolate DERUGE | 59→549 |
| [NGJJ01000798](https://www.ncbi.nlm.nih.gov/nuccore/NGJJ01000798) | CC1406-203 |  | 1177→1665 |
| [JQ695935](https://www.ncbi.nlm.nih.gov/nuccore/JQ695935) | unknown | unknown | 12→530 |
| [NGJJ01000573](https://www.ncbi.nlm.nih.gov/nuccore/NGJJ01000573) | CC1406-203 |  | 1→260 |
| [JAAVMX010000002](https://www.ncbi.nlm.nih.gov/nuccore/JAAVMX010000002) | IOZ07 |  | 18702095→18702586 |
| [NGJJ01000573](https://www.ncbi.nlm.nih.gov/nuccore/NGJJ01000573) | CC1406-203 |  | 13133→13623 |
| [AJ488254](https://www.ncbi.nlm.nih.gov/nuccore/AJ488254) |  | Isolate H1023 | 1←457 |
| [KT339190](https://www.ncbi.nlm.nih.gov/nuccore/KT339190) | Clone 2-7-13-14 | Clone 2-7-13-14 | 37→537 |
| [GU246288](https://www.ncbi.nlm.nih.gov/nuccore/GU246288) |  | Isolate ISO-21 | 6→497 |
| [GU246287](https://www.ncbi.nlm.nih.gov/nuccore/GU246287) |  | Isolate ISO-20 | 6→497 |
| [JAAVMX010000019](https://www.ncbi.nlm.nih.gov/nuccore/JAAVMX010000019) | IOZ07 |  | 6233→6733 |
| [KT232017](https://www.ncbi.nlm.nih.gov/nuccore/KT232017) |  | Clone 6-17-9 | 37→522 |
| [KT232018](https://www.ncbi.nlm.nih.gov/nuccore/KT232018) |  | Clone 6-17-10 | 37→522 |
| [AB067743](https://www.ncbi.nlm.nih.gov/nuccore/AB067743) |  | Isolate Yagyusan | 55→544 |
| [AB067744](https://www.ncbi.nlm.nih.gov/nuccore/AB067744) |  | Isolate Satoku | 54→544 |
| [KT720572](https://www.ncbi.nlm.nih.gov/nuccore/KT720572) |  | Isolate SANBI5308 | 11→324 |
| [KT218215](https://www.ncbi.nlm.nih.gov/nuccore/KT218215) | unknown | unknown | 11→324 |
| [KT232019](https://www.ncbi.nlm.nih.gov/nuccore/KT232019) |  | Clone 6-17-11 | 37→525 |
| [AB067739](https://www.ncbi.nlm.nih.gov/nuccore/AB067739) |  | Isolate Nyaramu-1 | 59→544 |
| [AB067740](https://www.ncbi.nlm.nih.gov/nuccore/AB067740) |  | Isolate Nyaramu-2 | 59→544 |
| [KT232028](https://www.ncbi.nlm.nih.gov/nuccore/KT232028) |  | Clone 6-17-20 | 37→525 |
| [KT232010](https://www.ncbi.nlm.nih.gov/nuccore/KT232010) |  | Clone 6-17-2 | 37→525 |
| [KT232012](https://www.ncbi.nlm.nih.gov/nuccore/KT232012) |  | Clone 6-17-4 | 37→525 |
| [MG770309](https://www.ncbi.nlm.nih.gov/nuccore/MG770309) | unknown | unknown | 1→301 |
| [GU246286](https://www.ncbi.nlm.nih.gov/nuccore/GU246286) |  | Isolate ISO9 | 6→497 |
| [KT339178](https://www.ncbi.nlm.nih.gov/nuccore/KT339178) |  | Clone 1-7-13-4 | 56→537 |
