# Supplementary material for: Altered GC- and AT-biased genotypes of Ophiocordyceps sinensis in the stromal fertile portions and ascospores of natural Cordyceps sinensis
Source: PLoS One. 2023 Jun 8;18(6):e0286865. doi: 10.1371/journal.pone.0286865 (PMC10249794; doi:10.1371/journal.pone.0286865)
Supplement: S3 Table — Note: Peak G represents GC-biased Genotype #1 H. sinensis; Peak A indicates AT-biased genotypes of O. sinensis (Fig 1). Peaks C and T denote 2 transversion mutation genotypes of unknown upstream and downstream sequences. “↑↑↑↑” denotes significant increases in intensity ratios greater than four-fold compared to that in the pre-ejection SFP. “―” means that one of the allelic peaks was missing and no ratio could be calculated. (DOCX) [file pone.0286865.s007.docx]

**S3 Table. Mass intensity ratios of the SNP peaks of transition and transversion mutation genotypes in the SFPs prior to and after ascospore ejection and of developmental failure.**

| **Extension primer** | **Allelic ratio** | **Intensity ratio** | | | |
| --- | --- | --- | --- | --- | --- |
|  |  | Pre-ejection SFP  (*cf*. S2.1 Fig) | Post-ejection SFP  (*cf*. S2.2 Fig) | SFP of developmental failure  (*cf*. S2.3 Fig) |  |
| 067721-531 | G:A | 1.06 (3.8÷3.6) | 19.3 **(↑↑↑↑**; 5.5÷0.3) | 13.5 (**↑↑↑↑**; 5.4÷0.4) |  |
|  | G:C | ― | ― | ― |  |
|  | G:T | 12.7 (3.8÷0.3) | ― | ― |  |

Note: Peak G represents GC-biased Genotype #1 *H. sinensis*; Peak A indicates AT-biased genotypes of *O. sinensis* (Fig 1). Peaks C and T denote 2 transversion mutation genotypes of unknown upstream and downstream sequences. “**↑↑↑↑**” denotes significant increases in intensity ratios greater than four-fold compared to that in the pre-ejection SFP. “―” means that one of the allelic peaks was missing and no ratio could be calculated.
