# Supplementary material for: Altered GC- and AT-biased genotypes of Ophiocordyceps sinensis in the stromal fertile portions and ascospores of natural Cordyceps sinensis
Source: PLoS One. 2023 Jun 8;18(6):e0286865. doi: 10.1371/journal.pone.0286865 (PMC10249794; doi:10.1371/journal.pone.0286865)
Supplement: S4 Table — Note: Peak T represents AT-biased Genotypes #5‒6 and #15 of O. sinensis, and Peak C indicates AT-biased Genotype #4 of O. sinensis (cf. Fig 1). Peak G was nearly flat and negligible. Peak A denotes a transversion mutation genotype of unknown upstream and downstream sequences. “↔” indicates no significant change (within 20% variation), and “↑” denotes increases in intensity ratios less than two-fold compared to that for the pre-ejection SFP. “―” means that one of the allelic peaks was missing, and no ratio could be calculated. (DOCX) [file pone.0286865.s008.docx]

## **S4 Table. Mass intensity ratios of the SNP allelic peaks of transition and transversion mutant genotypes in SFPs prior to and after ascospore ejection and of developmental failure.**

| **Extension primer** | **Allelic ratio** | **Intensity ratio** | | |
| --- | --- | --- | --- | --- |
|  |  | **Pre-ejection SFP**  (*cf*. S3.1 Fig) | **Post-ejection SFP**  (cf. S3.2 Fig) | **SFP of developmental failure**  (*cf*. S3.3 Fig) |
| 067740-328 | T:C | 14.1 (52.0÷3.7) | 16.5 (**↔**; 61.0÷3.7) | 19.3 (**↑**; 65.5÷3.4) |
|  | T:G | ― | ― | ― |
|  | T:A | 11.1 (52.0÷4.7) | 20.3 (**↑**; 61.0÷3.0) | 17.7 (**↑**; 65.5÷3.7) |

Note: Peak T represents AT-biased Genotypes #5‒6 and #15 of *O. sinensis,* and Peak C indicates AT-biased Genotype #4 of *O. sinensis* (*cf*. Fig 1). Peak G was nearly flat and negligible. Peak A denotes a transversion mutation genotype of unknown upstream and downstream sequences. “**↔**” indicates no significant change (within 20% variation), and “**↑**” denotes increases in intensity ratios less than two-fold compared to that for the pre-ejection SFP. “―” means that one of the allelic peaks was missing, and no ratio could be calculated.
