# Supplementary material for: Altered GC- and AT-biased genotypes of Ophiocordyceps sinensis in the stromal fertile portions and ascospores of natural Cordyceps sinensis
Source: PLoS One. 2023 Jun 8;18(6):e0286865. doi: 10.1371/journal.pone.0286865 (PMC10249794; doi:10.1371/journal.pone.0286865)
Supplement: S5 Table — Note: Peak G represents GC-biased Genotype #1 H. sinensis; Peak A indicates AT-biased genotypes of O. sinensis (cf. Fig 1). Peaks C and T denote 2 transversion mutation genotypes of unknown upstream and downstream sequences. “↔” indicates no significant change (within 20% variation) compared to the intensity ratios for fully ejected ascospores. “―” means that one of the allelic peaks was missing, and no ratio could be calculated. (DOCX) [file pone.0286865.s009.docx]

## **S5 Table. Mass intensity ratios of the SNP allelic peaks of transition and transversion mutant genotypes in fully and semi-ejected ascospores.**

| **Extension primer** | **Allelic ratio** | **Intensity ratio** | |
| --- | --- | --- | --- |
|  |  | Fully ejected ascospores  (Mass spectrum not shown) | Semi-ejected ascospores  (Mass spectrum not shown) |
| 067721-531 | G:A | 18.3 (5.5÷0.3) | 16.0 (**↔**; 8.0÷0.5) |
|  | G:C | ― | ― |
|  | G:T | ― | ― |

Note: Peak G represents GC-biased Genotype #1 *H. sinensis*; Peak A indicates AT-biased genotypes of *O. sinensis* (*cf*. Fig 1). Peaks C and T denote 2 transversion mutation genotypes of unknown upstream and downstream sequences. “**↔**” indicates no significant change (within 20% variation) compared to the intensity ratios for fully ejected ascospores. “―” means that one of the allelic peaks was missing, and no ratio could be calculated.
