# Supplementary material for: Altered GC- and AT-biased genotypes of Ophiocordyceps sinensis in the stromal fertile portions and ascospores of natural Cordyceps sinensis
Source: PLoS One. 2023 Jun 8;18(6):e0286865. doi: 10.1371/journal.pone.0286865 (PMC10249794; doi:10.1371/journal.pone.0286865)
Supplement: S6 Table — Note: Peak C represents AT-biased Genotype #5 of O. sinensis, and Peak T indicates other AT-biased genotypes of O. sinensis (cf. Fig 1). Peaks G and A denote 2 transversion mutation genotypes of unknown upstream and downstream sequences. “↑” indicates a significant increase in the intensity ratio less than two-fold compared to that of the fully ejected ascospores. “―” means missing one of the allele peaks, and no ratio could be calculated. (DOCX) [file pone.0286865.s010.docx]

## **S6 Table. Mass intensity ratios for the SNP allelic peaks for multiple transition and transversion mutation genotypes in the fully and semi-ejected ascospores.**

| **Extension primer** | **Allelic ratio** | **Intensity ratio** | |
| --- | --- | --- | --- |
|  |  | Fully ejected ascospores  (Mass spectrum not shown) | Semi-ejected ascospores  (Mass spectrum not shown) |
| 067740-328 | T:C | ― | ― |
|  | T:A | **14.0** (42.0÷3.0) | **22.0** (**↑**; 66÷3.0) |
|  | T:G | ― | ― |

Note: Peak C represents AT-biased Genotype #5 of *O. sinensis,* and Peak T indicates other AT-biased genotypes of *O. sinensis* (*cf*. Fig 1). Peaks G and A denote 2 transversion mutation genotypes of unknown upstream and downstream sequences. “**↑**” indicates a significant increase in the intensity ratio less than two-fold compared to that of the fully ejected ascospores. “―” means missing one of the allele peaks, and no ratio could be calculated.
