# Supplementary material for: Altered GC- and AT-biased genotypes of Ophiocordyceps sinensis in the stromal fertile portions and ascospores of natural Cordyceps sinensis
Source: PLoS One. 2023 Jun 8;18(6):e0286865. doi: 10.1371/journal.pone.0286865 (PMC10249794; doi:10.1371/journal.pone.0286865)
Supplement: Attachment — Submitted filename: 2022-10-27 response to the comment of Dr. Sauli.docx [file pone.0286865.s012.docx]

October 26, 2022

To:

Editor-in-chief

Professor Emily Chenette

*PLOS ONE*

Staff Editor

Dr. Jian-Hong Zhou

Academic Editor

Dr. Elingarami Sauli

RE:	Manuscript re-submission (PONE-S-22-35837 or PONE-D-22-28022), entitled:

**Altered GC- and AT-biased genotypes of *Ophiocordyceps sinensis* in the stromal fertile portions and ascospores** **of natural *Cordyceps sinensis***

By: Yu-Ling Li, Ling Gao, Yi-Sang Yao, Xiu-Zhang Li, Zi-Mei Wu, Zhou-Qing Luo, Wei-Dong Xie, Jian-Yong Wu, Jia-Shi Zhu

Dear Drs. Chenette, Zhou and Sauli:

Thank you for allowing us to resubmit our manuscript entitled “**Altered GC- and AT-biased genotypes of *Ophiocordyceps sinensis* in the stromal fertile portions and ascospores** **of natural *Cordyceps sinensis***” to PLOS ONE for consideration for publication. All authors agreed on the manuscript. Please send all paper correspondence related to the review and publication of this manuscript to me.

Our first submission (June 17; PONE-D-22-17417) of the manuscript was rejected because of suspicion “based on the fact that the authors seem to have divided related work into separate manuscripts”, assessed by Dr. Sauli in his decision letter on July 6. We submitted our appeal request to PLOS ONE:

“Indeed, the data in these 2 papers were from the same research project. However, the manuscript PONE-D-22-17339 contains 3 tables and 8 figures of 15 panels. And the manuscript PONE-D-22-17417 contains 9 tables and 9 figures of 28 panels. If we combine the 2 papers, there will be a total of 12 tables and 17 figures of 43 panels. It is extremely lengthy having so many tables and figures in a single paper. I decided to divide the work into 2 separate manuscripts because no journals will accept such a lengthy paper.

In addition, the manuscript PONE-D-22-17339 deals with qualitative analysis and the manuscript PONE-D-22-17417 deals with semi-quantitative analysis. We then submitted them as companion papers to PLOS ONE.

Now the manuscript PONE-D-22-17339 is in its revision stage. We hope that Dr. Sauli will reconsider his rejection decision towards our manuscript PONE-D-22-17417.”

Natural *Cordyceps sinensis* is a medicinal insect-fungi complex containing multiple *Ophiocordyceps sinensis* fungi and a dead larval body from the family Hepialidae. This resubmitted manuscript PONE-S-22-35837 (or PONE-D-22-28022) (first submission: PONE-D-22-17417) and the companion paper (PONE-D-22-17339) entitled “**Differential coexistence of multiple genotypes of *Ophiocordyceps sinensis* in the stromata, ascocarps and ascospores of natural *Cordyceps sinensis***” used different sets of molecular techniques to achieve different goals, the results of which will supplement and support each other. In addition to the examination of the microscopic morphology of natural *C. sinensis* and the phylogeny of multiple *O. sinensis* genotypes, the companion paper PONE-D-22-17339 uses fungus- and genotype-specific primers and cloning-based amplicon sequencing techniques to profile the co-occurrence of multiple genotypes of *O. sinensis* fungi in different compartments of natural *C. sinensis* in different maturational stages.

The resubmitted paper PONE-S-22-35837 (or PONE-D-22-28022) (first submission: PONE-D-22-17417) focuses on the sexual life of *O. sinensis* and examines multiple genotypes of *O. sinensis* fungi in the ascospores and stromal fertile portion (SFP) that is densely covered with ascocarps of natural *C. sinensis*. This paper uses the biochip-based single nucleotide polymorphism MALDI-TOF mass spectrometry technique and a stratification genotyping strategy to monitor dynamic alterations of multiple *O. sinensis* mutants in a semiquantitative manner. The evidence of dynamic alterations of *O. sinensis* genotypes indicates that the metagenomic members in the SFPs and ascospores of natural *C. sinensis* differentially play symbiotic roles in the sexual life of *O. sinensis*. Rectifying the traditional view that natural *C. sinensis* is a static, single-fungus product, this study provides critical information assisting in the design and artificial cultivation of *C. sinensis* to supplement the increasingly scarce natural resources of the *C. sinensis* insect-fungi complex, which is in the list of the second grade of endangered natural products.

We have revised our manuscript PONE-S-22-35837 (or PONE-D-22-28022) since last submission (PONE-D-22-17417), largely based on the relevant peer-review comments to the companion paper (PONE-D-22-17339), which is in its revision stage.

We include a newly published review paper and a special note as a reference of this resubmission on the academic confusion in regard to usages of different Latin names because of the reviewers’ concern toward the companion paper (PONE-D-22-17339). The Latin name confusion has existed >170 years since the 1840s. We are not taxonomists and are not able to make a taxonomic decision on the academic confusion. Taxonomists in mycology and botany-TCM (traditional Chinese medicine) reached a consensus on natural *C. sinensis* ≠ *O. sinensis* fungus/fungi, and there is a need to give a new name to the natural insect-fungi complex. However, they have not reached a consensus on Latin name usage for natural *C. sinensis* but instead stand strongly in support of their own rename suggestions.

The second issue of confusion relates to implementation of the IMA’s nomenclature rule of “One Fungus=One Name” in *O. sinensis* research. A group of mycologists suggested implementing the nomenclature rule by discarding the anamorphic name *Hirsutella sinensis* and replacing it with the teleomorphic name *O. sinensis*. However, this suggestion appears to be inappropriate and inapplicable because “One Fungus” is the prerequisite of the nomenclature rule and because many culture-dependent and independent mycological studies have proven that the Latin name *O. sinensis* very likely represents a group of *O. sinensis* fungi. Both our papers, PONE-S-22-35837 (or PONE-D-22-28022) (last submission: PONE-D-22-17417) and PONE-D-22-17339, confirm the literature findings and the conclusion that there are multiple teleomorphic *O. sinensis* fungi. These findings lead us to be extremely careful in the usage of different Latin names in our manuscripts, which must be based on scientific evidence and follow the suggestions from taxonomists in mycology and TCM. The rationale of our Latin name usages is listed at the end of the special note.

The data presented in this manuscript and the companion paper greatly contribute to the scientific knowledge regarding natural *C. sinensis* and *O. sinensis* fungi. Hence, we believe that this manuscript (PONE-S-22-35837 or PONE-D-22-28022), as well as the companion paper (PONE-D-22-17339), is appropriate for publication in PLOS ONE because of the new findings of the microscopic morphology, phylogenetics and molecular biology examinations and because of the research approaches and strategies used in this study to answer complex scientific questions for natural multifungal products. This paper should therefore be of broad interest to your readership.

Best regards,

Jia-Shi Zhu, Ph.D.

Telephone: +1 (858) 705-3789

E-mail:	[zhujosh@163.com](mailto:zhujosh@163.com)
