# Supplementary material for: Altered GC- and AT-biased genotypes of Ophiocordyceps sinensis in the stromal fertile portions and ascospores of natural Cordyceps sinensis
Source: PLoS One. 2023 Jun 8;18(6):e0286865. doi: 10.1371/journal.pone.0286865 (PMC10249794; doi:10.1371/journal.pone.0286865)
Supplement: Attachment — Submitted filename: 2023-5-11 Responses to Reviewer#1.docx [file pone.0286865.s013.docx]

**Responses to the comments from Reviewer #1**

Reviewer #1’s comments:

The authors described genotypes of Ophiocordyceps sinensis - insect-fungal complex. The work is novel. It is based on solid experimental background. Though it is hard to read due to redundancy in technical details, and not clear overall presentation. As I understand, the text was revised and reorganized after previous submission. It has an accompanied paper that complements the O. sinensis study.

However, the manuscript is still too large. Figures from 5 to 9 are very similar. May move them to a Supplement as well as other redundant text.

The presentation is not clear for reader with common biological educational background. It missed important details on the object of the study.

The reference style is not appropriate – bulk citations everywhere! It should be not more than 2-3 references together in the text. Otherwise need a phrase, add details, cite the references separately.

See - [6‒10], [ 7‒25] and multiple other similar in-text citations ([6‒16,19‒21,26‒38]).

The Abstract is unclear. It should have some description of the object (it is given only later in the main text).

The ‘Objective’ of the Abstract should be extended to state the problem – it is rather about insect-fungal interactions and association with genotypes. The problem seems be to narrow (only about “SFP densely covered with numerous ascocarps and ascospores”?

Line 44: “cultivated in our laboratory (altitude 2,200)” – need details about laboratory, is it unique place or could be cultivated anywhere at same altitude? What is the laboratory?

Line 54: “mutation alleles of unknown sequences” – unclear, rephrase

Line 64: the keywords could be more precise, exhaustive, and not so long. Comment on MassARRAY (it is not in the text).

Citations are in bulk. Please cite separately, add details, new sentences.

Even in the phrase (line 74) “Modern pharmacological studies … [2-5]” need name the studies, what is modern and recent?

Line 77: “≠” – this sign is from mathematics. Explain by words about the terminology, that it differs, or not equal.

General short names “C. sinensis” might be mixed with tea plant Latin name. It is worthy to repeat all the designation in this area again, not use new abbreviations.

Line 89: “[ 7‒25]’ such citation is not appropriate. Give necessary details, add a phrase. Or remove redundant references if not used further in the text.

Line 99: “widely accepted because the evidence meets the first and second criteria of Koch’s postulates” – please name the postulate, give a reference. Phrase “widely accepted” should be confirmed.

Line 104: “Many studies…” – name these studies. The references at the end of the sentence again are redundant ([6‒ 10,13,15‒16,37,47‒54]).

Line 111: “Genotypes #1 [28,30,32,39], #3 [55‒56], #4…” - it is not clear what this terminology means (#1, #2..) – is it commonly accepted, or designed by the authors? Some new figure with map of geographic origins would be helpful. And some description of geographic and environment conditions.

Line 117: “[61 (the companion paper)]” – such citation is not appropriate. May refer to this paper by other way, not using complex parentheses.

Line 134: Section ‘Reagent; could be after section ‘Collection…’ in the text. Logically it should be after Methods description.

Line 140: MassARRAY – should be commented about the tool, complex abbreviation.

Line 144: “were purchased in local markets” – need details – condition, location of the markets, how stable such specimens. Where the specimens were collected before, transported?

Line 151: “(cf. Fig 3A below)” – the figures numbering should start from 1. 1,2, then 3. Please rearrange.

Line 155: “Fig 1 of the companion paper [61])” – again it is not clear. Refer first on the figures in the same text. May add some references to a Supplement to the paper, or other published paper. It is just not clear for reader.

Line 159: “The humidity..” – need give exact values.

Line 195: “The primers Hsprp1, Hsprp3, ITS4, P1, P2 and P4…” – these nomenclature is unclear. Need comment first, provide details about known genes and primers.

Figure 1 should be as an additional figure. First should be information about the object, known genotypes. Might be some photos as in Fig.3

Now Figure 1 gives not information about AT and GC content.

Line 207: “Genotypes #1‒3 and #13‒14 are GC-biased..” – it is not visible.

Line 215: “GenBank” – need exact link to access the data in Genbank Details of phylogenetic analysis are too common (and too many just bioinformatics references). It is better to describe known strains.

Line 252: “iLEX Gold cocktail” – need add details.

Figure 3 is good, but has too many panels. Please make it more compact to fit to one page. Separate Figure 3 onto smaller figures.

Figures 4,5,6,7,8,9 are very similar. May keep one-two figures in the main text, and more other figures to a Supplement (new Supplementary material file, or to online resource)

Discussion section is rather technical, and it has again redundant references. The section (starts from line 807) “True causative fungus/fungi of natural C. sinensis” is more interesting. That part of text could be in the beginning of paper. Conclusion section is rather sort.

The dynamic alterations of genotypes is hypothesized, but not proved in current study.

Could keep it as the hypothesis. It needs lager statistics and a greater number of specimens to prove anyway.

The reference list is too long, as well as overall paper size. It could be reduced without loss of information but make presentation more clear and convenient. I suggest reformatting the text, remove up to 20% of reference, remove repeated figures with MS plots (or place it to a Supplementary file)

Authors’ responses to the comments from Reviewer #1:

[1] Reviewer #1’s comment “it is hard to read due to redundancy in technical details, and not clear overall presentation”.

Authors’ response:

This comment is very general. We tried to reduce the technical details and to make our presentation clearer. We have been using the English language editing service provided by editors of American Journal Experts (AJE). The manuscript has been edited by the AJE editors several times (June 3, 2022, September 27, 2022, October 22, 2022) prior to submission to PLOS ONE. The revised manuscript has been reedited again by the editors of American Journal Experts (May 7, 2023).

[2] Reviewer #1’s comment “the manuscript is still too large. Figures from 5 to 9 are very similar. May move them to a Supplement as well as other redundant text”

“Figures 4,5,6,7,8,9 are very similar. May keep one-two figures in the main text, and more other figures to a Supplement (new Supplementary material file, or to online resource)”.

Authors’ response:

This paper presents semiquantitative analyses of dynamic alterations of GC- and AT-biased genotypes of *O. sinensis* in the stromal fertile portions (SFPs with ascocarps) and ascospores of natural *Cordyceps sinensis* using the MassARRAY SNP mass spectrometry genotyping technique in addition to the histology examination of the SFP samples. This semiquantitative study is different from what is presented and published in the companion paper ([Ref 31] Li et al. PLOS ONE 2023, 18(3): e0270776. <https://doi.org/10.1371/journal.pone.0270776>), which deals with qualitative data and answers “yes or no” (fungal presence or absence) questions. [Ref 31] revealed the differential co-occurrence of several genotypes of *O. sinensis* and other interspecific fungi.

Based on the findings of the companion paper [Ref 31], we designed a stratification genotyping strategy by using several elongation primers for SNP analyses and performed Biochip-based SNP MALDI-TOF mass spectrometry genotyping using the 384-well SpectroCHIP and MassARRAY genotyping technique provided by Sequenom Company, which was acquired later by Agena Bioscience Company. This technique has been widely used to genotype the fungal components in the stroma of natural *Cordyceps sinensis* and in many other studies. However, the results presented in these papers were in Chinese:

[Ref 19 in Chinese] Gao et al. 2011 [J. Peking Univ. (Health Sci.). 2011;43(2): 259–266, PMID: 21503123 [Doi: 10.3969/i.issn.1671-167X.2011.02.019](http://xuebao.bjmu.edu.cn/CN/abstract/abstract10193.shtml)].

[Ref 20 in Chinese] Gao et al. 2012 [J. Peking Univ. (Health Sci.). 2012;44(3): 454–463, PMID: 22692321 [Doi: 10.3969/i.issn.1671-167X.2012.03.025](http://xuebao.bjmu.edu.cn/CN/abstract/abstract10419.shtml)].

Figs 4‒9 in the previous submission show the mass spectra, presenting the key findings of semiquantitative analyses. Figs 4‒7 are the SNP mass spectra for the stromal fertile portion (SFP) that is densely covered with numerous ascocarps, and Figs 8‒9 are the SNP mass spectra for the 2 types of ascospores of natural *Cordyceps sinensis*. The mass spectra essentially show up to 4 allelic peaks, representing nucleotides A, C, G, and T in the DNA sequences of the samples. Although some of the mass spectra look similar to some degree, they show changes in the allelic peaks and allelic ratios and demonstrate dynamic changes in the *O. sinensis* genotypes in different samples of the SFPs and the 2 types of ascospores of natural *Cordyceps sinensis*. This is a typical molecular physiology approach.

Natural *Cordyceps sinensis* was previously believed to be a single-fungal natural product [Refs 32,38‒40]. However, mycobiota studies reported by mycologists demonstrated that the stroma and caterpillar body of natural *Cordyceps sinensis* contain >90 fungal species from at least 37 genera with different predominate fungi [Refs 15‒16]. Metagenomic and metatranscriptomic studies confirmed the co-occurrence of multiple fungi in the compartments of *Cordyceps sinensis*. Molecular mycology studies detected 17 genotypes of *O. sinensis* in different combinations in the *Cordyceps sinensis* compartments [Refs 8‒23,31]. Bushley et al. [Ref 50] demonstrated that *Cordyceps sinensis* hyphae and ascospores are multicellular heterokaryons with mono-, bi-, tri-, and tetra-nucleated structures. The various heterokaryotic cells of the *C. sinensis* hyphae and ascospores likely contain different fungal genetic materials [Ref 64]. The component fungi grow at varying growth rates in different compartments in different maturational phases [Refs 14,19‒20]. Accordingly, Prof. Zong-Qi Liang concluded that natural *Cordyceps sinensis* is an integrated microecosystem with a complex lifecycle and that each fungal member plays a different role(s) in the *Cordyceps sinensis* compartments in different maturational phases [Ref 29]. The current paper ([PONE-D-22-28022) aims to explore for the first time the dynamic alterations of multiple genotypes of *O. sinensis* in SFPs with ascocarps and ascospores of natural *Cordyceps sinensis*. The data presented in the current paper are different from those presented in the companion paper [Ref 31], which answered “yes-or-no” (fungal presence or absence) questions by exploring the co-occurrences of multiple genotypes of *O. sinensis* in the immature and mature stromata, SFPs with ascocarps, and ascospores of natural *Cordyceps sinensis*.

Because of the co-occurrence of several GC- and AT-biased genotypes in the SFPs and ascospores, simple SNP analysis is not able to differentiate the individual genotypes in the complex natural samples. We thus designed a stratifying strategy using different SNP elongation primers and genotyped the samples in a stratified manner. This research strategy utilizes the natural chemistry technical skill in our SNP molecular study, which, we believe, is one of the defining points of our paper but may be unfamiliar to some mycologists, who have not read our previous relevant publications in Chinese [Refs 21‒22,58].

We first used the elongation primers 067721-477 and 067721-531 to detect co-occurrence of the GC- and AT-biased genotypes. The templates of the second-step SNP elongation PCR were the amplicons of the first-step PCR, although the primers used in the first-step PCR preferentially amplified the GC-biased Genotype #1 and produced more GC-biased copies than the AT-biased counterparts.

Using the stratification strategy, we then used the elongation primer 067740-328 to detect the extremely low quantity of AT-biased Genotype #4 in all SFP samples of *Cordyceps sinensis,* as shown in Fig 6 of the previous submission (now Supplementary S3 Fig in revision). The mass spectra of Figs 6A‒6C (now Supplementary S3.1‒S3.3 Figs) show essentially the same topology, which demonstrated very important evidence, *i.e.*, the consistency of the extremely low quantity of Genotype #4 in all SFP samples, providing important information and constituting a solid base for the next step of stratification analysis.

In the second step of stratification analysis, we used the elongation primer 0677444-324 to identify AT-biased Genotypes #6 and #15, differentiating them from AT-biased Genotype #5 (Fig 7 of the previous submission; now Fig 5 in the revision).

Thus, Figs 4‒7 and related Tables 3‒6 (now Figs 4‒5, Tables 3‒4, and Supplementary S1‒S4 Figs and S3‒S4 Tables) are not redundant. Each of the figures and tables demonstrates unique findings using different extension primers and different SFP samples under the stratification study design, presenting different semiquantitative genotyping results.

Although Figs 4‒9 demonstrate unique findings and help our readers understand the whole story and the dynamic alterations of several genotypes of *O. sinensis* in the SFPs and ascospores, we have taken Reviewer #1’s advice to reduce the size of the main paper.

(1) We moved Fig 5 (including Panels 5A, 5B, 5C, and 5D) and Table 4 of the original submission to a supplementary file, which are now Supplementary S2 Fig (including Panels S2.1, S2.2, S2.3, and S2.4) and S3 Table in the revision.
(2) We moved Fig 6 (including Panels 6A, 6B, and 6C) and Table 5 to the supplementary file, which are now Supplementary S3 Fig (including Panels S3.1, S3.2, and S3.3) and S4 Table in the revision. Although Fig 6 in the previous submission (now Supplementary S3 Fig in the revision) showed similar SNP mass spectrometry topologies for all SFP samples, it demonstrates a very important finding, *i.e.*, the extremely low quantity of AT-biased Genotype #4 in all SFP samples, which is crucial to the stratification analysis plan of the whole project.
(3) We moved the mass spectra for the SFP of developmental failure to the supplementary file. Panels 4C and 7C of Figs 4 and 7 in the original submission are now Supplementary S1 Fig and S4 Fig in the revision, respectively.
(4) We moved Tables 8‒9 related to the ascospore analyses to the supplementary file, which are now Supplementary S3‒S4 Tables, respectively.
[3] Reviewer #1’s comments “**The reference style** is not appropriate – **bulk citations** everywhere! It should be not more than 2-3 references together in the text. Otherwise need a phrase, add details, cite the references separately”;

“**See - [6‒10], [7‒25]** and multiple other similar in-text citations ([6‒16,19‒21,26‒38])”;

“**Citations are in bulk**. Please cite separately, add details, new sentences”;

“**Line 89: “[7‒25]’** such citation is not appropriate. Give necessary details, add a phrase. Or remove redundant references if not used further in the text.”;

“**Line 104**: “Many studies…” – name these studies. The references at the end of the sentence again are redundant ([6‒ 10,13,15‒16,37,47‒54])”;

“**Line 117**: “[61 (the companion paper)]” – such citation is not appropriate. May refer to this paper by other way, not using complex parentheses”:

“The reference list is too long, as well as overall paper size. It could be reduced without loss of information but make presentation more clear and convenient. I suggest reformatting the text, remove up to 20% of reference, remove repeated figures with MS plots (or place it to a Supplementary file)”.

Authors’ response:

We have revised the citations in the revision by adding separate and detailed descriptions with only a few citations.

We have deleted many references. The reference list has been reduced from 81 references to 67 references.

[4] Reviewer #1’s comments “The Abstract is unclear. It should have some description of the object (it is given only later in the main text)”.

“The ‘Objective’ of the Abstract should be extended to state the problem – it is rather about insect-fungal interactions and association with genotypes. The problem seems be to narrow (only about “SFP densely covered with numerous ascocarps and ascospores”?”

Authors’ response:

We have revised the objective of the Abstract to:

“**Objective:** To examine multiple genotypes of *Ophiocordyceps sinensis* in a semiquantitative manner in the stromal fertile portion (SFP) densely covered with numerous ascocarps and ascospores of natural *Cordyceps sinensis* and to outline the dynamic alterations of the coexisting genotypes in different developmental phases.”

The current paper only studied samples of “SFP densely covered with numerous ascocarps and ascospores” of natural *Cordyceps sinensis* and addresses the dynamic alteration of several genotypes of *O. sinensis* in these samples using a semiquantitative approach.

As we described in the paper and responded above, the dynamic alterations of several genotypes of *O. sinensis* in the stromata and caterpillar bodies of *Cordyceps sinensis* during maturation have been studied previously. Several related studies have been published using different sets of techniques.

(1) Using Southern blotting and restriction fragment length polymorphism (RFLP) plus cloning-based amplicon sequencing techniques after restrictive endonuclease digestion:

[Ref 19 in English] Zhu et al. 2010 [Am. J. Biomed. Sci. 2010;2(3): 217–238. doi: 10.5099/aj100300217]

[Ref 23 in Chinese] Yao et al. 2011 [J. Fungal Res. 2011;9(1): 37–49,53. DOI：10.13341/j.jfr.2011.01.007]

[Ref 20 in Chinese] Zhu et al. 2012 [J. Fungal Res. 2012;10(2): 100–112. DOI：10.13341/j.jfr.2012.02.007]

(2) Using the biochip-based MassARRAY MOLD-TOF mass spectrometry SNP genotyping technique:

[Ref 21 in Chinese] Gao et al. 2011 [J. Peking Univ. (Health Sci.). 2011;43(2): 259–266. [Doi: 10.3969/i.issn.1671-167X.2011.02.019](http://xuebao.bjmu.edu.cn/CN/abstract/abstract10193.shtml)].

[Ref 22 in Chinese] Gao et al. 2012 [J. Peking Univ. (Health Sci.). 2012;44(3): 454–463. [Doi: 10.3969/i.issn.1671-167X.2012.03.025](http://xuebao.bjmu.edu.cn/CN/abstract/abstract10419.shtml)].

The companion paper [Ref 31] published qualitative data on the co-occurrence of multiple genotypes and other interspecific fungi in SFPs with ascocarps and ascospores of *Cordyceps sinensis*. With a slightly different study objective, the current study presents semiquantitative SNP genotyping of several genotypes of *O. sinensis* to explore the dynamic alterations of the *O. sinensis* genotypes in the SFPs prior to and after ascospore ejection, SFPs of developmental failure, and 2 types of ascospores.

Reviewer #1 commented “it is rather about insect-fungal interactions and association with genotypes”. We believe that the formation of SFPs with ascocarps and the production and ejection of ascospores are signs of full maturity of *Cordyceps sinensis* in the stage of *O. sinensis* sexual reproduction. The samples of SFPs and ascospores contain only fungal materials, which are very different from the caterpillar body of *Cordyceps sinensis* that contains both larval tissues and fungal materials. Thus, the current study does not address “insect-fungal interactions”.

[5] Reviewer #1’s comment “Line 44: “cultivated in our laboratory (altitude 2,200)” – need details about laboratory, is it unique place or could be cultivated anywhere at same altitude? What is the laboratory?”

Authors’ response:

We have provided the longitude and latitude of the laboratory in the revision. The laboratory is on the Qinghai-Tibet Plateau (altitude 2,254 m; longitude and latitude of 101°45’ E, 36°40’ N).

Natural *Cordyceps sinensis* may mature earlier in the production areas of low latitudes than in circumpolar latitude areas because of the ecology in the *Cordyceps sinensis* production areas, including different strengths of sunshine, temperature, rains, plateau breeze, etc. The collection of mature *Cordyceps sinensis* with a long stroma and an expanded stromal fertile portion (SFP) close to the tip of the stroma (*cf*. Fig 3.1) was performed in natural fields; however, continuing cultivation of mature *Cordyceps sinensis* in the laboratory for further maturation of SFPs and ascospores does not have to be in the location of our laboratory. The important factor is the high altitudes, where sunshine is much stronger than at low altitudes and the oxygen partial pressure is low, favoring continuing maturation of the SFPs and ascospores of mature *Cordyceps sinensis* specimens.

[6] Reviewer #1’s comment “Line 54: “mutation alleles of unknown sequences” – unclear, rephrase”.

Authors’ response:

SNP studies identify single nucleotide polymorphisms, and the SNP mass spectra show polymorphisms at a single nucleotide position using a particular elongation primer. The elongation primers were designed based on sequence alignments with the sequences of transition mutants (*cf*. Fig 1), aiming to detect and differentiate GC- and AT-biased transition genotypes in the current study. At the same time, we needed to rule out any possible interference of known transversion mutation genotypes of *O. sinensis* through sequence alignments [Refs 9‒11]. However, we realize that theoretically, there may be some unknown transversion point mutation genotypes yet to be identified, either interindividual genotypes or repetitive copies of ITS sequences co-occurring in an *O. sinensis* genome.

Thus, when the SNP mass spectra detect nucleotide alleles at a single nucleotide position using an elongation primer that we designed for the current study, we know the upstream and downstream sequences of the single transition allele(s) but do not know the upstream and downstream sequences of the transversion mutation allele(s).

The advantage of this technique for genotyping is precisely and highly efficiently detecting a single nucleotide or nucleotide polymorphism at a single nucleotide position. The disadvantage is unknown upstream and downstream sequences of the transversion mutation alleles under the experimental design of the current study.

It is thus important to have the companion paper [Ref 31, Li et al. PLOS ONE 2023, 18(3): e0270776], which has been published and deals with mutant sequences qualitatively. However, the qualitative analysis of the sequences of several *O. sinensis* genotypes in the companion paper [Ref 31] was not capable of quantitatively monitoring the dynamic changes of the mutant genotypes, while the semiquantitative analysis in the current paper supplements the companion paper [Ref 31] and monitors the dynamic alterations of the genotypes.

Line 54 (now Line 56) in the abstract has been revised to “Mass spectra also showed transversion mutation alleles of unknown upstream and downstream sequences”. We also rephrased similar sentences in the main text.

[7] Reviewer #1’s comment “Line 64: the keywords could be more precise, exhaustive, and not so long. Comment on MassARRAY (it is not in the text)”.

“Line 140: MassARRAY – should be commented about the tool, complex abbreviation.”

Authors’ response:

One of the Keywords: “MassARRAY single nucleotide polymorphism MALDI-TOF mass spectrometry genotyping” is rather long. Unfortunately, it is indeed a long technical term, even without spelling out the abbreviation “MALDI-TOF”. There is a section in the “Materials and Methods” describing the technology and experimental method of MassARRAY SNP MALDI-TOF MS genotyping. We have cited a 2001 PNAS paper [Ref 54] for the >20-year-old technology.

“MassARRAY®” is the trademark of a MALDI-TOF mass spectrometry platform for both the mass spectrometry analyzer and the operating technology. The MassARRAY analyzer and the technology were developed by Sequenom Company, which was acquired later by Agena Bioscience Company.

This part of the examinations was contracted out and performed by CapitalBio Corporation in Beijing, China. This technique uses a 384-well biochip (SpectroCHIP of Sequenom), and samples are run in a MassARRAY MALDI-TOF mass spectrometry analyzer to detect SNP polymorphisms for genotyping.

We have shortened the term in the Keywords to: “SNP MALDI-TOF mass spectrometry genotyping”.

For your reference, there are 12 places mentioning the trademark “MassARRAY” in the text.

[8] Reviewer #1’s comment “Even in the phrase (line 74) “Modern pharmacological studies … [2-5]” need name the studies, what is modern and recent?”

Authors’ response:

We have revised the sentences: “The therapeutic profiles of *Cordyceps sinensis* for respiratory, cardiovascular, endocrine, kidney, and liver systems, etc., have been studied using modern pharmacological approaches and summarized [2‒3]. The lifespan-extending properties of natural *Cordyceps sinensis* and its mycelial fermentation products were validated in mice in a 4-year pharmacology study, and the molecular mechanisms have been explored using a whole-gene expression biochip technique [4‒5].”

The term “Modern pharmacological studies” is a methodology term relative to traditional TCM “pharmacological” methodology. *Cordyceps sinensis* is a superb TCM therapeutic agent and has many therapeutic functions in several organ systems, as summarized in Refs 2‒3, which were published in 1998 and summarized hundreds of papers and results from a variety of study designs. TCM uses historical (vertical) experience accumulation methods, *i.e.*, evidence-based methods, to explore the therapeutic effects and potencies of natural “herbal” products and herbal formulae. Modern pharmacological studies use experimental methods in animals and/or in humans with randomized, parallel comparisons using positive drug-control pairs and/or placebo-control pairs. Differences in methodology are obvious between “modern” and traditional “pharmacological” approaches.

The therapeutic functions have been reexamined using “Modern pharmacological” methodologies since the 1980s, which cannot be called “recent” studies.

[9] Reviewer #1’s comment “Line 77: “≠” – this sign is from mathematics. Explain by words about the terminology, that it differs, or not equal.”

Authors’ response:

We have revised it to “*i.e.*, natural *Cordyceps sinensis* is not equal to the *O. sinensis* fungus”.

[10] Reviewer #1’s comment “General short names “C. sinensis” might be mixed with tea plant Latin name. It is worthy to repeat all the designation in this area again, not use new abbreviations.”

Authors’ response:

We agree. The “abbreviation” of [*Camellia sinensis*](https://en.wikipedia.org/wiki/Camellia_sinensis) is also *C. sinensis*, and *Clonorchis sinensis* can be abbreviated *C. sinensis*. To address the suggestion from Reviewer #1, we have revised our manuscript by replacing the abbreviation *C. sinensis* after the first appearance of the full Latin name in the paper with the full name, *Cordyceps sinensis*. However, we disagree that *C. sinensis* is a “new abbreviation” of *Cordyceps sinensis*.

We hope that the Academic Editor of the paper and PLOS ONE office and production team will agree with this practice with the revised usage of the Latin name.

[11] Reviewer #1’s comment “Line 99: “widely accepted because the evidence meets the first and second criteria of Koch’s postulates” – please name the postulate, give a reference. Phrase “widely accepted” should be confirmed.”

Authors’ response:

We have revised the sentence: “The sole *O. sinensis* anamorph hypothesis for *H. sinensis* [32] has been widely accepted because the evidence meets the first and second criteria of Koch’s postulates.”

Adding significantly more references to back up the phrase “widely accepted” is not an issue. However, doing so will conflict with another of Reviewer #1’s comments: “The reference list is too long … I suggest reformatting the text, remove up to 20% of reference”. Fortunately, because “widely accepted” is synonymous with common knowledge, we may not need to list so many references in our paper to back up the phrase “widely accepted”.

[12] Reviewer #1’s comment “Line 111: “Genotypes #1 [28,30,32,39], #3 [55‒56], #4…” - it is not clear what this terminology means (#1, #2...) – is it commonly accepted, or designed by the authors? Some new figure with map of geographic origins would be helpful. And some description of geographic and environment conditions”.

Authors’ response:

This is a long story of scientific discovery and genetic and phylogenetic analysis.

The multiple genotypes (#1, #4‒5) of *O. sinensis* were first reported by mycologists Kinjo and Zang 2001 [Ref 37], although Ingeborg B. Engh (1999) discovered mutant Genotype #4 earlier in his or her graduate study at the University of Oslo, Norway (unpublished data). The Norwegian–US mycology group Stensrud et al. 2005 (Mycol. Res. 2005;109: 41−56) disclosed the sequence (AJ786590 of Genotype #4) and uploaded it to GenBank.

Because of the confusion regarding many mutants under the same name *Cordyceps sinensis* (renamed *Ophiocordyceps sinensis* by Sung et al. Stud Mycol, 2007, 57(1), 5‒59), the Norwegian group Stensrud et al. 2007 [Ref 55] conducted a phylogenetic study using a Bayesian algorithm and recognized for the first time the 3 groups of *Cordyceps sinensis* mutants under the same Latin name. They grouped the mutants as GC-biased Group A (*i.e.*, Genotype #1) and AT-biased Groups B‒C (*i.e.*, Genotypes #4‒5), as well as Groups D‒E for other interspecific fungi, and proposed that the mutants formed a species complex. They believed that these *Cordyceps sinensis* mutants were genetic offspring evolved through accelerated evolution processes from a common genetic ancestor.

Medicinal mycologists Yang et al. 2008 and Xiao et al. 2009 [Refs 12‒13] at the Institute of Materia Medica of the Chinese Academy of Medical Sciences further studied *Cordyceps sinensis* mutants (Groups A‒C, *i.e.*, Genotypes #1, #4‒5) and reported the inconsistent co-occurrence of the mutants (genotypes) in different compartments of natural *Cordyceps sinensis* specimens in different maturation stages collected from remote production areas. The authors rejected the “cryptic species” hypothesis proposed by Stensrud et al. 2007 [Ref 55] and instead believed that the sequences of mutant genotypes reside in independent fungal genomes, belonging to interindividual *O. sinensis* fungi. In addition to the mutants in Groups A‒C (*i.e.*, Genotypes #1, #4‒5), the authors also discovered another AT-biased mutant (Genotype #6) of *O. sinensis* and uploaded its sequence (EU555436) to GenBank.

Mycologists Zhang et al. 2009 [Ref 46] (corresponded by Prof. Xing-Zhong Liu, one of the former presidents of the Chinese Mycological Association and the Asian Mycological Association) at the Institute of Microbiology of the Chinese Academy of Sciences reported another mutant sequence of *O. sinensis*, which exhibited GC bias and was identified in 2 *Cordyceps sinensis* specimens collected from Nyingchi, Tibet. However, the genetic characteristics of this mutant containing multiple transition point mutations are distinct from those of GC-biased Group A (Genotype #1) and AT-biased Groups B‒C (Genotypes #4‒5). The authors then hypothesized that Nyingchi of Tibet is the center of origin for *O. sinensis* and established a fungal evolutionary geography for *O. sinensis*. Zhang et al. 2009 uploaded the mutant sequences (FJ654148 and FJ654149) to GenBank. The mutant sequences were later labeled as GC-biased Genotype #3 of *O. sinensis*.

An Indian mycology group identified a mutant sequence (MW990119) of *O. sinensis* from a *Cordyceps sinensis* specimen (CBUS3) collected in Yumesamdong (27°84'88" N 88°69'04" E), North Sikkim ([Biswa](https://www.researchgate.net/profile/Vashkar-Biswa-2?_sg%5B0%5D=s64P5ZUVJ1URLNv-HWGqXciQYtExpw0K0Gn04x-eFkcYYbujtfmeglPR1RT6_vo7j_dVx7k.BZFOnsdLnJAnlA-_98ww6o_l2e0RZTbrEoZOGIVIXr800AnIgJVtOfGgeZRdGzs-CeiiR8JWsiKntqSseqrGcQ&_sg%5B1%5D=gH4fbS1jxaDGoMKtgMckCpKZgl6NmQo-caHVz3XmXaCWZbnsxiZaE8emVME05LttkcptTSM.1qyaUHrooLPNhrshHIjvEYiDVj3yij3Ir_l6b_UdI3HJs7PSyQbd2yhbQb6WePLT9Ds93M-3w_zEBKlSA48_zQ) et al. 2021, Indian J Hill Farming 2021;34: 268‒275). The collection location is far (approximately 500 km) from Nyingchi in Tibet (29°38′56″ N, 94 21′41″ E). MW990119 is 99% homologous to the sequence HM595984 of the representative sequence of GC-biased Genotype #3 but only 95% similar to AB067721 of Genotype #1 *H. sinensis*. The discovery of sequence MW990119 confirmed the natural occurrence of Genotype #3 of *O. sinensis* but may suggest that the “center of origin” hypothesis for Nyingchi of Tibet and the “fungal evolutionary geography” for *O. sinensis* species may need to be reconsidered or reanalysized.

Molecular biologists Zhu et al. 2010, 2012 [Refs 19‒20] discovered another GC-biased mutant (Genotype #2) of *O. sinensis* co-occurring with GC-biased Genotype #1 *H. sinensis* in the stromata of immature, maturing, and mature *Cordyceps sinensis*. This mutant genotype features multiple scattered transition point mutations. The 2 co-occurring GC-biased genotypes (#1 and #2) showed dynamic alterations in an asynchronized, disproportional manner in the stromata of *Cordyceps sinensis* during maturation (Figure R1), indicating their genomic independence, belonging to interindividual *O. sinensis* fungi.

### *Figure R1. Dynamic alterations of the abundance of the amplicons of Genotypes #1 and #2 of O. sinensis in the stromata of C. sinensis during maturation (modified from Fig 6 of [Ref 16]). Immature C. sinensis had a very short stroma of 1.5 cm. Maturing C. sinensis had a stroma of 4.0 cm without an expanded fertile portion close to the stroma tip. Mature C. sinensis had a long stroma of 7.0 cm and showed the formation of an expanded fertile portion close to the stromal tip, which was densely covered with ascocarps.*

Aggregating the genotyping information for the *O. sinensis* mutants, Zhu et al. 2012 [Ref 20] summarized *O. sinensis* mutants of at least 6 genotypes, all of which contain multiple scattered transition point mutations. Although *O. sinensis* genotypes were labeled Groups A‒C in the phylogenetic study by Stensrud et al 2007 [Ref 55], and later by Zhang et al. [2013] (J Indust Microbiol Biotechnol 2013; 40: 365–378), the sequences of other interspecific fungi were labeled sequentially as Groups D‒E, leaving no room for new discoveries of *O. sinensis* genotypes, such as GC-biased Genotypes #2‒3 and AT-biased Genotype #6 at that time. With no alternatives, Zhu et al. 2012 [Ref 20] had to switch the alphabetical grouping system to a numeric system, and the sequential numbers are only for *O. sinensis* genotypes (*cf*. Supplementary S1 Table), not for other interspecific fungi. This sequential numeric grouping system for *O. sinensis* genotypes has been used in many publications.

**GC-biased Genotypes #1‒3:** (*cf*. Supplementary S1 Table)

Group A was renamed Genotype #1 of *O. sinensis* (*H. sinensis*), represented by AB067721.

Genotype #2 of *O. sinensis* was discovered by Zhu et al. 2010, 2012, and is represented by MG770309.

Genotype #3 of *O. sinensis* was discovered by Zhang et al. 2009, and is represented by HM595984.

**AT-biased Genotypes #4‒6:** (*cf*. Supplementary S1 Table)

Group B was renamed Genotype #4 of *O. sinensis*, represented by AB067744.

Group C was renamed Genotype #5 of *O. sinensis*, represented by AB067740.

Genotype #6 of *O. sinensis* was first discovered and the sequence was uploaded to GenBank as an uncultured endophytic fungus (EU555436) by Xiao and Zhu 2009. Genotype #6 is represented by KJ720572 (*O. sinensis*), which was discovered later. The GenBank database contains at least 4 sequences of Genotype #6.

After Zhu et al. 2012 [Ref 20], mycologists Zhang et al. 2013 (J Indust Microbiol Biotechnol 2013; 40: 365–378) conducted a GenBank data mining study involving >500 ITS sequences under the name of *O. sinensis*, followed by phylogenetic analysis. They confirmed several phylogenetic clades similar to those previously published by Stensrud et al. 2007 [Ref 55], *i.e.*, Clades A‒C of *O. sinensis*, Clades D‒E for other interspecific fungi, and additional Clades F‒G for other fungi. Similar to Stensrud et al. 2007 [Ref 55], Zhang et al. 2013 did not leave room for newly discovered mutants between *O. sinensis* clades (Clades A‒C) and other interspecific fungal clades (Clades D‒G).

In contrast to the homogenous GC-biased Group A sequences containing only Genotype #1 reported by Stensrud et al. 2007 [Ref 55], Zhang et al. 2013 reported the quite divergent GC-biased Clade A, containing the sequences of GC-biased Genotype #1 *H. sinensis* and many other variable GC-biased sequences (including the Genotype #3 sequence). These variable sequences showed long phylogenetic distances to GC-biased Genotype #1, while the sequence of GC-biased Genotype #2 had not been uploaded to GenBank at that time.

Based on the phylogenetic findings by Zhang et al. 2013, these variable GC-biased sequences were further analyzed in 2015 through 2022 [Refs 8‒11] and found to contain multiple scattered transversion and insertion/deletion point mutations. These sequences were genetically and phylogenetically different from the transition point mutation genotypes (Genotypes #1‒6 of *O. sinensis*) and then grouped as Genotypes #7‒12 based on the number of transversion and insertion/deletion alleles and the allelic ratio of insertion/deletion alleles *vs*. transversion point mutations.

**Genotypes #7‒12 are GC-biased:** (*cf*. Supplementary S1 Table)

Genotype #7 of *O. sinensis* was detected in the stroma of a *Cordyceps sinensis* specimen collected in Qinghai Province by Chen et al. 2011 [Ref 48], represented by AJ488254. Interestingly, a Genotype #1 sequence (AJ488255) was detected in the caterpillar body of the same specimen without co-occurrence of the Genotype #7 sequence.

Genotype #8 of *O. sinensis* was uploaded by an Indian group, represented by GU246286.

Genotype #9 of *O. sinensis* was uploaded by an Indian group, represented by GU246288.

Genotype #10 of *O. sinensis* uploaded by an Indian group, represented by GU246287.

Genotype #11 of *O. sinensis* was uploaded by an Indian group, represented by JQ695935.

Genotype #12 of *O. sinensis* was uploaded by an Indian group, represented by GU246296.

**Genotypes #8‒12** were discovered and uploaded to GenBank by Indian scholars, but no full publications could be found.

The companion paper [Ref 31] discovered additional variable sequences of *O. sinensis* (*cf*. Figs 2,6‒7 and Tables 2‒3 of Ref 31; and Supplementary S1 Table of this study).

Genotypes #13‒14 are GC-biased (*cf*. Ref 31 and Supplementary S1 Table of this study), featuring differential reciprocal substitutions of DNA segment sequences and genetic material recombination between genomes of 2 parental fungi, Groups A Genotype #1 *H. sinensis* (AB067721) and an Group E AB067719-type fungus.

Genotypes #15‒17 are AT-biased (*cf*. Ref 31 and Supplementary S1 Table of this study), containing multiple scattered transition point mutations.

The above response to Reviewer #1’s comment shows the discovery history in detail and the identification, phylogenetic clustering, and renaming of the *O. sinensis* genotypes from the alphabetical grouping system to the numeric system. Many other papers and books have published the multiple *O. sinensis* genotypes, using the alphabetical or numeric system.

Of course, academic debates also exist in regard to the variable *O. sinensis* mutants or genotypes, such as hypotheses of a sole anamorph and sole teleomorph of *O. sinensis* [Ref 32], multiple teleomorphs of *O. sinensis* [Refs 31,40], nomenclature of *O. sinensis* using Genotype #1 *H. sinensis* strain as the reference strain [Sun et al. 2007; Stud Mycol 2007; 57: 5–59], cryptic species [Ref 55], independent species [Ref 13], genomically independent genotypes [Refs 6‒11,19‒23,31], the species paradox between anamorphic inoculants and the sole teleomorph of cultivated *Cordyceps sinensis* [Ref 40], ITS pseudogenes [Ref 49], repeat-induced point mutations (RIP) (Li et al. 2020, IMA Fungus 2020;11: 18), GC-biased repetitive copies of ITS sequences [Ref 57], and pros and cons to the hypotheses in many papers.

Nevertheless, the sequences of Genotypes #2‒17 do not reside in the genome assemblies of the 5 *H. sinensis* (Genotype #1 of *O. sinensis*) Strains 1229, CC1406-203, Co18, IOZ07, and ZJB12195 [Refs 41‒45] and cannot be pseudogenic components of the *H. sinensis* genome [Refs 7‒11,57; and Zhu et al. 2019 Mol. Phylogenet Evol. 2019;139: 106322. doi: 10.1016/j.ympev.2018.10.034; and Li et al. 2020 Mol. Phylogenet. Evol. 2020;146: 106728. <https://doi.org/10.1016/j.ympev.2019.106728>]. The *H. sinensis* genome assemblies do contain multiple repetitive copies of ITS sequences. However, these ITS repeats are GC-biased and genetically and phylogenetically distinct from AT-biased genotypes [Ref 11,57; *cf*. Fig 2 of this paper]. The bottom line is that the discoveries of Genotypes #1‒17 have been documented in many papers, and the genotypic sequences reside in independent fungal genomes and belong to interindividual naturally existing *O. sinensis* fungi.

[13] Reviewer #1’s comment “Line 134: Section ‘Reagent; could be after section ‘Collection…’ in the text.”

Authors’ response:

We have moved the sections accordingly, although this comment seems to reflect a personal preference.

[14] Reviewer #1’s comment “Line 144: “were purchased in local markets” – need details – condition, location of the markets, how stable such specimens. Where the specimens were collected before, transported?”

Authors’ response:

We have provided information regarding the “locations of the markets” with latitude-longitude and altitude information in that section.

In regard to the question “Where the specimens were collected before transported?”, there were 2 sets of specimens, as we stated in that section.

(1) Some of the *Cordyceps sinensis* specimens were used for histological examination, as shown in Fig 3. The specimens were purchased in local markets from local farmers who collected the specimens in nearby production areas. The specimens underwent thorough surface disinfection after purchase and were kept frozen in liquid nitrogen or dry ice during transport and storage in our laboratory prior to further processing, as described in the Materials and Methods section.

The *Cordyceps sinensis* specimens in superb condition were selected one-by-one before purchase in the markets. This was crucial during specimen purchase to ensure the success of our study.

(2) Other mature *Cordyceps sinensis* specimens were used for continuing cultivation in our laboratory, as described in the Materials and Methods section, for sampling the SFP tissues before and after ascospore ejection and SFPs of developmental failure and for collecting the 2 types of ascospores. The samples of SFPs and ascospores were used in the SNP genotyping.

To purchase these specimens, we followed the local farmers to nearby production fields and watched them harvest *Cordyceps sinensis* specimens with the outer mycelial cortices and soil surrounding the caterpillar body. Of course, these specimens were in superb condition, allowing successful continuing cultivation in our laboratory. The specimens were replanted in paper cups in soil obtained from *C. sinensis* production areas and cultivated in our laboratory, as shown in Fig 1 of Ref 31 and reproduced in Supplementary SR1 Fig.

[15] Reviewer #1’s comment “Line 151: “(cf. Fig 3A below)” – the figures numbering should start from 1. 1,2, then 3. Please rearrange.”

Authors’ response:

We have changed the figure numbering, although this seems to be a personal preference. BTW, our PLOS ONE paper just published used figure numbering with a number+letter system, such as Figs 3(A), 3(B), 3(C), etc.

[16] Reviewer #1’s comment “Line 155: “Fig 1 of the companion paper [61])” – again it is not clear. Refer first on the figures in the same text. May add some references to a Supplement to the paper, or other published paper. It is just not clear for reader.”

Authors’ response:

We have changed the reference citation to Fig 1 of [31] in revision (Ref 61 in last submission). In addition, we took Reviewer #1’s advice and reproduced Fig 1 of [61] (now Ref 31 in revision) in the supplementary file as Supplementary SR1 Fig:

Mature *Cordyceps sinensis* specimens were cultivated in paper cups in our Xining laboratory (altitude of 2,254 m) as shown in Supplementary SR1(A) Fig. The fully ejected ascospores were collected using double layers of autoclaved weighing papers as shown in Supplementary SR1(B) Fig. Numerous semi-ejected ascospores adhered to the outer surface of an ascus during the massive ejection of ascospores, as shown in Supplementary SR1(C) Fig.

[17] Reviewer #1’s comment “Line 159: “The humidity..” – need give exact values.”

Authors’ response:

The humidity of our laboratory during continuing cultivation of mature *Cordyceps sinensis* was maintained by spraying water using an atomizer twice a day in the morning and evening [61]. We revised the sentence to “The relative humidity of our laboratory was maintained at approximately 30%‒40%, and the absolute humidity on the surface of *Cordyceps sinensis* stromata was maintained at up to 90%‒100% by spraying water using an atomizer twice a day in the morning and evening [31].”

[18] Reviewer #1’s comment “Line 195: “The primers Hsprp1, Hsprp3, ITS4, P1, P2 and P4…” – these nomenclature is unclear. Need comment first, provide details about known genes and primers.”

Authors’ response:

The primers were designed, named, tested previously, and used in previous publications. We added the references [Refs 14,19‒23]. The sequences and locations of the “primers Hsprp1, Hsprp3, ITS4, P1, P2 and P4” are given in Table 1 and Fig 1 of the current paper. “these nomenclature” of primers has been documented previously in different papers, and we have no intention of changing the primer names in this paper.

[19] Reviewer #1’s comment “Figure 1 should be as an additional figure. First should be information about the object, known genotypes. Might be some photos as in Fig.3.”

“Now Figure 1 gives not information about AT and GC content.”

“Line 207: “Genotypes #1‒3 and #13‒14 are GC-biased” – it is not visible.”

Authors’ response:

Fig 1 shows not only the sequence alignment of multiple transition mutants (genotypes of *O. sinensis*) but also the sequences, locations, and directions of SNP elongation primers. Sequence alignment information is essential for designing the primers in the current study.

Fig 1 is repeatedly referred to in the Results section when displaying each of the mass spectrometry tracings in Figs 4‒9 (now Figs 4‒7 and Supplementary S1‒S4 Figs in the revision). To fully understand the study design and each of the SNP allelic peaks in the SNP mass spectrometry tracings and their relevance to the quantitative measures, readers may need to cross-check back and forth the sequence alignment and the sequences, locations, and directions of SNP elongation primers shown in Fig 1. Thus, we believe that moving Fig 1 to the supplementary file would sacrifice the integrity and coherence of the paper and reduce the readability of the paper and readers’ interest. It is clear that keeping Fig 1 in the main paper is appropriate to facilitate readers’ understanding of the study design and results of the paper.

Among the *O. sinensis* genotypes shown in Fig 1, Genotypes #1‒2 and #13‒14 are GC-biased genotypes, and Genotypes #4‒6 and #15‒17 are AT-biased genotypes. The terms “GC- and AT-biases” and “GC- and AT-biased” were first published for *O. sinensis* mutants (genotypes) by the Norwegian mycologists Stensrud et al. 2007 [Ref 55], followed by many publications [Refs 6‒13,19‒23,31.49,55,57‒59], based on the genetic and phylogenetic results for the mutants. With limited information about the *O. sinensis* mutants at that time, Stensrud et al. 2007 named GC-biased Genotype #1 as Group A and AT-biased Genotypes #4‒5 as Groups B‒C, followed by Groups D‒E for other interspecific fungi.

Regarding Reviewer #1’s comments about the “AT and GC content” of the genotypes and “Genotypes #1‒3 and #13‒14 are GC-biased – it is not visible”, we added Supplementary S1 Table in the supplementary file, providing information on the AT and GC contents of the 17 genotypes of *O. sinensis*. The AT contents were 39.1%±3.1% for the GC-biased genotypes (Genotypes #1‒3 and #7‒14) and 48.9%±1.7% for the AT-biased genotypes (Genotypes #4‒6 and #15‒17) (P<0.001).

[20] Reviewer #1’s comment “Line 215: “GenBank” – need exact link to access the data in Genbank Details of phylogenetic analysis are too common (and too many just bioinformatics references). It is better to describe known strains.”

Authors’ response:

Regarding the first part of the comment, ““GenBank” – need exact link to access the data in Genbank”, it seems that Reviewer #1 is asking us to provide GenBank hyperlinks to the sequences analyzed in Fig 2.

Fig 2 shows a maximum likelihood (ML) phylogenetic tree. This phylogenetic analysis was contracted out to Nanjing Genepioneer Biotechnologies Co., along with conferring a Bayesian majority-rule consensus tree [Ref 57] using the same set of sequence data. We received photo files for the 2 trees, which are not editable with the addition of hyperlinks to the sequences in the GenBank database. Alternatively, we added a Supplementary S2 Table in the supplementary file with hyperlinks to the GenBank database.

The second part of the comment “Details of phylogenetic analysis are too common (and too many just bioinformatics references). It is better to describe known strains” seems to be a personal preference regarding the appearance of the phylogenetic tree. The detailed sequence information had to be retained in Fig 2 for the following reasons.

First, many of the sequences analyzed in Fig 2 do not have strain information. Some were obtained from natural *Cordyceps sinensis* specimens or isolates through direct PCR sequencing or amplicon cloning-based sequencing. Thus, listing only strain information in Fig 2 is not possible.

Second, many GenBank sequences contain partial 18S and partial 28S sequences, which are upstream and downstream of the complete ITS1, 5.8S, and ITS2 segment sequences, respectively. We then had to delete the partial 18S and 28S segments before analysis of ITS1-5.8S-ITS2 sequences. Thus, including sequence ranges is necessary to provide detailed and correct information on exact sequence segments for phylogenetic analysis.

Third, genome assemblies of *H. sinensis* Strains CC1406-203 and IOZ07 contain multiple repetitive ITS copies, as shown in Fig 2 and Supplementary S2 Table. The genome of Strain CC1406-203 contains at least 7 repetitive ITS copies, and the genome of Strain IOZ07 contains at least 17 repetitive ITS copies. Not listing the sequence ranges for the segments of repetitive ITS copies would make the figure chaotic, and then listing all the names of the genome assemblies, strain names or isolate names or clone names, species information, and sequence ranges would be necessary.

Most importantly, Fig 2 listing the detailed sequence information shows the same phylogenetic topology for the genotype clades compared with the phylogenetic trees that were conferred using Bayesian and many other phylogenetic clustering algorithms and published in many prior publications [Refs 7‒13,31.37,40,49,55,57]. All these phylogenetic trees support and confirm each other.

[21] Reviewer #1’s comment “Line 252: “iLEX Gold cocktail” – need add details.”

Authors’ response:

“iLEX Gold cocktail” is a commercial reagent product of Sequenom (the company was later acquired by Agena Bioscience Company) and specifically formulated for use with the 384-well biochip (SpectroCHIP of SEQUENOM) and MassARRAY MALDI-TOF mass spectrometry analyzer. Our genotyping project using MassARRAY SNP MALDI-TOF mass spectrometry technology was contracted out to a service company, CapitalBio Corporation in Beijing, China. Unfortunately, the detailed formula of the commercial cocktail is unavailable. However, according to the reference paper [Ref 54; Buetow et al. 2001, Proc. Natl. Acad. Sci. USA. 2001;98(2): 581–584] and the website information of Agena Bioscience Company, the “iLEX Gold cocktail” should contain routine PCR reagents (polymerase, dNTPs, etc.) and dideoxy A, T, C, and G terminator nucleotides (ddNTPs) to control DNA chain elongation. This information has been added to the Reagents section.

[22] Reviewer #1’s comment “Figure 3 is good, but has too many panels. Please make it more compact to fit to one page. Separate Figure 3 onto smaller figures.”

Authors’ response:

We have followed the figure instructions of PLOS journals and used the recommended PACE system to adjust the resolution of figures to 300 PPI. The current sizes of the figures are the results of the PACE modification system. We believe that the technical team or production team of PLOS ONE journal will adjust the size of the figures to fit them to one page, as Reviewer #1 requested.

[23] Reviewer #1’s comment “Discussion section is rather technical, and it has again redundant references. The section (starts from line 807) “True causative fungus/fungi of natural C. sinensis” is more interesting. That part of text could be in the beginning of paper. Conclusion section is rather sort.”

Authors’ response:

The Conclusion section has been rewritten.

We agree that the discussion section is technical. We have worked on the “redundant references” following the reviewer’s suggestion.

Reviewer #1 is interested in “True causative fungus/fungi of natural C. sinensis”, which has been a focus of *Cordyceps sinensis* research. To date, that part of the research has been nonconclusive, very complex, and controversial. Thus, we are hesitant to put more information in this regard in the Introduction section. Moreover, the “True causative fungus/fungi” issue is not the main focus of the current paper but a topic that should be discussed in the Discussion section because Li et al. 2020 (Sci Chin (Life Sci) 2020, 63(7): 1093–1095. https://doi.org/10.1007/s11427-020-1686-7) reported using the *Cordyceps sinensis* ascospores as the inoculant in the successful industrial cultivation project.

For your reference, we are providing the following relevant information here:

Hu et al. 2013 [Ref 41] (corresponded by one of the former presidents of the Chinese Mycological Association and coauthored by another former president of the Chinese Mycological Association) reported the injection of a mycelial mixture of *H. sinensis* Strains Co18 and QH195-2 into the second proleg of 40 larvae of *Hepialidae* sp. However, this inoculation approach failed to induce the development of sexual fruiting bodies and ascospores. The authors cited 2 other studies that failed to induce production of the fruiting body and ascospores of *Cordyceps sinensis* in laboratory settings:

(1) Holliday and Cleaver. A review. Medicinal value of the caterpillar fungi species of the genus Cordyceps (Fr.) Link (Ascomycetes). Intl J Med Mushrooms, 2008;10: 219–234.
(2) Stone R. Bhutan. Improbable partners aim to bring g biotechnology to a Himalayan kingdom. Science, 2010;327: 940–941).

Zhang et al. 2013 [Ref 63] (corresponded by one of the former presidents of the Chinese Mycological Association) summarized >40 years of mycology experience with failed induction of fruiting body and ascospore production in laboratory settings, either in fungal cultures or after infecting insects with fungal inoculants.

Wei et al. 2016 [Ref 40] (corresponded by a senior member of the Chinese Academy of Sciences and the President Emeritus of the Chinese Mycological Association) reported industrial success of artificial cultivation of *C. sinensis*. However, the authors presented definite species mismatches between the inoculants (3 GC-biased anamorphic *H. sinensis* strains reportedly in the paper) and the teleomorph of *O. sinensis* (AT-biased Genotype #4) in the fruiting body of final cultivated *C. sinensis* and between the teleomorphs (GC-biased Genotype #1 and AT-biased Genotype #4 of *O. sinensis*) of natural and cultivated *C. sinensis*, respectively, while the sequences of Genotypes #1 and #4 of *O. sinensis* reside in the independent fungal genomes, belonging to interindividual *O. sinensis* fungi.

Li et al. 2016 [Ref 58] conducted inoculation/infection experiments using either conidia or mycelia of *H. sinensis*, ascospores of *Cordyceps sinensis*, or a combination of 2 wild-type fungal complexes (CH1 and CH2) as inoculants on *Hepialus armoricanus* larvae (n=100 larvae per inoculant experiment). Strains CH1 and CH2 were isolated from the living intestines of healthy larvae of *Hepialus lagii* and shared the same morphological and growth characteristics with those of *H. sinensis* but contained GC-biased Genotype #1 *H. sinensis* and AT-biased Genotypes #4‒6 of *O. sinensis* and *Paecilomyces hepiali*. The inoculation experiments demonstrated extremely low infection rates (1.4%‒3.5%) when the inoculation was performed with the conidia or mycelia of *H. sinensis* or the ascospores of natural *Cordyceps sinensis* but significantly higher infection rates (55.2±4.3%; P<0.001) when the inoculation was performed with the mixture of wild-type Strains CH1 and CH2 on the larvae of *H. armoricanus*.

Li et al. (2020) (corresponded by a senior member of the Chinese Academy of Sciences, the President Emeritus and one of the former presidents of the Chinese Mycological Association) published a Letter-to-Editor (Li W. et al. Sci Chin (Life Sci) 2020;63(7): 1093‒1095) in regard to the successful industrial cultivation of *C. sinensis*. The authors reported that the inoculant was the ascospores of natural *Cordyceps sinensis*, which is different from the first report using 3 *H. sinensis* strains by Wei et al. 2016 [Ref 40]. The authors named the ascospores of natural *C. sinensis* as *O. sinensis* in the paper. However, Li et al. 2023 [Ref 31, PLOS ONE 2023;18(3): e0270776. <https://doi.org/10.1371/journal.pone.0270776>] reported that *Cordyceps sinensis* ascospores contain GC-biased Genotype #1 *H. sinensis* and several AT-biased genotypes of *O. sinensis* and *Paecilomyces hepiali*. Coincidentally, Li et al. 2023 [Ref 31] indirectly confirmed that the inoculant in the successful industrial *Cordyceps sinensis* cultivation process used by Li et al. (2020) was a fungal complex containing GC-biased Genotype #1 *H. sinensis* and several AT-biased genotypes of *O. sinensis* and *P. hepiali* rather than a single *H. sinensis* fungus.

There are more interesting studies in this direction, and the results are nonconclusive, controversial, and debatable.

[24] Reviewer #1’s comment “The dynamic alterations of genotypes is hypothesized, but not proved in current study”

“Could keep it as the hypothesis. It needs lager statistics and a greater number of specimens to prove anyway”.

Authors’ response:

Our current study provides additional lines of evidence of “dynamic alterations of genotypes” of *O. sinensis*, which add to the preexisting evidence pool, and the evidence is much more than just a “hypothesis”.

Our current paper is not the first to elucidate the “dynamic alterations of genotypes” of *O. sinensis*. Xiao et al. 2009 [Refs 13], Zhu et al. 2010, 2012 [Refs 19‒20], Gao et al. 2011, 2012 [Refs 21‒22] and Li et al. 2023 [Ref 31] previously reported evidence of “dynamic alterations of genotypes” of *O. sinensis* in the immature, maturing, and mature stromata of natural *Cordyceps sinensis* using different research approaches. Our current study provides additional evidence of “dynamic alteration” of *O. sinensis* genotypes in the SFPs (with ascocarps) and ascospores of natural *Cordyceps sinensis*, supplementing the missing pieces of the puzzle.

We provide some of the published results here.

(1). Zhu et al. 2010 [Ref 19]: The ITS sequences of the GC-biased genotypes contained an *Eco*RI endonuclease cleavage site (G^▼^AATTC, in purple in Fig 1) at the position of nucleotides 294→299 of AB067721 (a representative sequence of Genotype #1 *H. sinensis*). The *Eco*RI cleavage site was lost in all AT-biased genotypes (GAATTT) due to a single-base C-to-T (cytosine→thymine) transition. To examine dynamic alterations of the natural biomasses of GC- and AT-biased genotypes of *O. sinensis* without PCR manipulation that causes nonlinear, exponential amplification of DNA sequences, the Southern blotting technique was applied to examine genomic DNA after enzymolysis with endonucleases *Ava*I, *Dra*I, and *Eco*RI [Zhu et al. 2010, Ref 19]. The upper panel of Figure R2 below shows the Southern blot using an *H. sinensis*-specific probe that was designed based on the ITS1 sequence of Genotype #1 *H. sinensis*:

**Figure R2. Southern blot of C. sinensis rDNA in the stroma and caterpillar body of natural C. sinensis during maturation.** (Zhu et al. 2010; Ref 19). Genomic DNA was isolated from the stroma or caterpillar body of premature (Pre) or mature (M) natural C. sinensis collected from Kangding County of Sichuan Province in China and prepared using the restriction enzymes EcoRI, DraI and AvaI. Upper panel: Using an H. sinensis-specific probe for Southern blotting. Lower Panel: Using a nonspecific 18S internal control probe for blotting. Caterpillar refers to the caterpillar body of natural C. sinensis.

a) The *O. sinensis* genotypes identified in the stromata: the left 2 lanes of the upper panel of Figure R2 show that the slower-migrating DNA moieties (*Eco*RI-resistant AT-biased genotypes) predominate naturally without PCR amplification in the stromata of immature (IM) and mature (M) *Cordyceps sinensis*. The faster-migrating moieties (*Eco*RI-sensitive GC-biased *H. sinensis*) are always minor moieties in the stromata during *Cordyceps sinensis* maturation.
b) The *O. sinensis* genotypes identified in the caterpillar bodies: although the second lane from the right in the upper panel of Figure R2 seems to show a single *Eco*RI-sensitive moiety in the immature (IM) caterpillar body but a doublet in the mature (M) caterpillar body shown in the far right lane, PCR experiments using genotype-specific primers and touch-down and nested PCR protocols were able to identify *Eco*RI-resistant AT-biased genotypes in the immature caterpillar body. These results suggest that the natural quantity of *Eco*RI-resistant AT-biased genotypes in the immature caterpillar body was below the detection limit of the Southern blotting technique, which is much less sensitive than PCR-based techniques. The quantity of AT-biased genotypes was dramatically and disproportionally increased from a microquantity in the immature caterpillar body to a level comparable to that of *Eco*RI-sensitive GC-biased *H. sinensis* in the mature caterpillar body.
c) The *O. sinensis* genotypes identified in the stroma and caterpillar body of immature *C. sinensis*: the first and third lanes from the left in Figure R2 show that the abundances and density ratios of *Eco*RI-resistant AT-biased genotypes and *Eco*RI-sensitive GC-biased *H. sinensis* differed significantly in the stroma and caterpillar body of immature *Cordyceps sinensis*, which were sampled from the same specimens.
d) The *O. sinensis* genotypes identified in the stroma and caterpillar body of mature *Cordyceps sinensis*: the first and third lanes from the right in Figure R2 show that the abundances and density ratio of *Eco*RI-resistant and *Eco*RI-sensitive genotypes differed in the stroma and caterpillar body of mature *Cordyceps sinensis*, which were sampled from the same specimens.

The disproportional distribution of GC- and AT-biased *O. sinensis* genotypes in the stromata and caterpillar bodies of natural *Cordyceps sinensis* and their asynchronous, disproportional alterations of natural biomasses without PCR amplification during *Cordyceps sinensis* maturation indicate that the sequences of AT-biased *O. sinensis* genotypes do not reside in the single genome of Genotype #1 *H. sinensis*. Instead, the genomically independent AT-biased *O. sinensis* genotypes are interindividually accompanied by GC-biased Genotype #1 counterpart in the stroma and caterpillar body of natural *Cordyceps sinensis*.

(2). Zhu et al. (2012, Ref 20) and Li et al. (2023, Ref 31): GC-biased Genotypes #1 and #2 coexisted in the immature, maturing, and mature stromata of natural *Cordyceps sinensis*. Their amplicon abundance was low in the immature stroma (1.0‒2.0 cm in stromal height). Genotype #1 showed a greatly elevated abundance in the maturing stroma (4.0‒4.5 cm in height without an expanded fertile portion close to the stromal tip), and its abundance then plateaued in the mature stroma (6.5‒7.0 cm in height, showing the formation of an expanded fertile portion close to the stromal tip, which is densely covered with numerous ascocarps) (Figure R1 above). In contrast to the maturation pattern of Genotype #1, the abundance of Genotype #2 remained at a low level in the maturing stroma and increased markedly in the mature stroma to a level that exceeded the abundance of Genotype #1. The co-occurrence of Genotypes #1 and #2 in the stromata with distinct maturation patterns indicates the genomic independence of the 2 GC-biased genotypes, which is proven by the absence of Genotype #2 sequences in the whole-genome sequences of 5 Genotype #1 *H. sinensis* strains [Refs 41‒45].

Li et al. 2023 [Ref 57] demonstrated that the genome of GC-biased Genotype #1 contains multiple repetitive ITS copies, but their genetic and phylogenetic characteristics are different from those of Genotypes #2‒17, providing additional evidence to confirm the interindividual co-occurrence of GC-biased Genotypes #1‒2 with dynamic alterations in natural *Cordyceps sinensis* during maturation.

3). Gao et al. [2011, 2012, Refs 21‒22] showed more evidence of “dynamic alterations” of GC- and AT-biased genotypes of *O. sinensis* using a MassARRAY SNP mass spectrometry genotyping approach.

To avoid individual differences in the samples studied, the DNA samples examined in these studies were individually extracted from 5‒10 samples, and equal quantities of the DNA extracts were combined prior to the study examinations.

We agree that all these results regarding “dynamic alterations of genotypes” of *O. sinensis* need to be re-examined on a broad scale with “lager statistics” since the samples examined in the above studies were collected from the *Cordyceps sinensis* production areas in Sichuan and Qinghai Provinces. Geographic differences may exist. We welcome more *Cordyceps* research groups actively involved in similar or different quantitative or semiquantitative research to prove or disprove, support or nonsupport the results published previously and the concept of “dynamic alterations of genotypes” of *O. sinensis* from different scientific perspectives.

Now let us return to the current paper using a semiquantitative SNP mass spectrum genotyping approach. The evidence for the “dynamic alterations of genotypes” of *O. sinensis* shown in the current paper includes the following:

First, to avoid individual differences in samples, we examined the SFP samples (1) prior to ascospore ejection, (2) after ascospore ejection, and (3) of developmental failure, each of which was represented by a combination of 5 *Cordyceps sinensis* specimens. The ascospores (4) fully ejected and (5) semiejected were collected from 5 *Cordyceps sinensis* specimens [Ref 31].

Second, Fig 4 and Table 3 show the intensities of transversion mutant alleles. Peaks C and T are dramatically altered relative to Peak A and G from pre- to postejection SFPs.

Third, Supplementary S2 Fig and S3 Table show that the intensities of Peaks G and A are dramatically altered from pre- to postejection SFPs.

Fourth, Fig 5 and Table 4 show that the intensities of Peaks T and C are dramatically altered from pre- to postejection SFPs.

Fifth, Fig 7 and Table 6 show that the intensities of Peaks T and C are dramatically altered from fully and semiejected ascospores.

Thus, from the previous studies and current study, the “dynamic alterations of genotypes” of *O. sinensis* in the stromata, caterpillar bodies, SFPs with ascocarps and ascospores of natural *Cordyceps sinensis* are supported by solid research evidence, which is much more than just a “hypothesis”. Again, we welcome more *Cordyceps* research groups actively engaged in similar or different quantitative or semiquantitative research to prove or disprove, support or nonsupport the results published previously and currently and the concept of “dynamic alterations of genotypes” of *O. sinensis* using different scientific approaches.
